# Elevation of secondary metabolites synthesis in *Brassica campestris* ssp. *chinensis* L. via exogenous inoculation of *Piriformospora indica* with appropriate fertilizer

**DOI:** 10.1371/journal.pone.0177185

**Published:** 2017-05-11

**Authors:** Muhammad Khalid, Danial Hassani, Muhammad Bilal, Jianli Liao, Danfeng Huang

**Affiliations:** 1 School of Agriculture and Biology, Shanghai Jiao Tong University, Shanghai, P.R. China; 2 State Key Laboratory of Microbial Metabolism, School of Life Sciences and Biotechnology, Shanghai Jiao Tong University, Shanghai, China; Henan Agricultural University, CHINA

## Abstract

This work evaluated the impact of exogenous soil inoculation of beneficial fungal strain *Piriformospora indica* on phytochemical changes and the related genes expression of Chinese cabbage (*Brassica campestris* ssp. *chinensis* L.) by greenhouse pot experiments. High performance liquid chromatography (HPLC) affirmed that among the different combinations of fungal and organic fertilizer treatments, the phenolic acids and flavonoids were considerably enriched in organic fertilizer and fungi (OP) followed by organic fertilizer, biochar, fungi (OBP) treated plants. The antiradical activity was higher in OP (61.29%) followed by P (60%) and organic fertilizer (OF) (53.84%) inoculated plants which positively correlated with chlorophyll, carotenoids and flavonoids level (P<0.05). Furthermore, results showed that the exogenous application of *P*. *indica* significantly (*P*<0.05) enhanced plant growth, as well as stimulating the activation of chlorophyll, carotenoids and other antioxidant related pathways. The RT-qPCR analysis indicated that key *FLS* gene triggering the synthesis of kaemferol was up-regulated by the inoculation of *P*. *indica*. In conclusion, the results revealed that organic fertilizer and *P*. *indica* (OP) is the most appropriate combination for improving phytochemical and antiradical properties in Pakchoi.

## Introduction

The consumption of fruits and vegetables could increase the human innate immunity against chronic diseases [1, 2]. The phytoconstituents including polyphenols, quercetin and flavonoids are largely demonstrated as important antioxidants and exhibit profound radical scavenging capabilities [3–7]. Chinese cabbage, which belongs to the *Brassicaceae* family is a predominantly consumed green leafy vegetable in China. It has the noteworthy health-promoting properties due to its high contents of fibers and phytochemicals [8].

The quality of fresh vegetables could be assessed based on their nutritional value, growing conditions and usage of fertilizer. Despite the fact that the genetic modification and agronomic manipulation methods are widely used to improve the nutritional value of plants, the inadequate public acceptance and soil specificity of genetically modified food are still the challenges. Alternatively, the modification of fertilization level brings a suitable method to improve the quality of edible plants, particularly in phytochemicals. To date, the use of beneficial microorganisms has become the sole alternative solution to ensure nutrient use efficiency and future food security because of the environmental concerns regarding excess utilization of chemical fertilizer. In general, the microorganisms exert positive effects on the growth characteristics by developing a holistic and functional relationship with plants [9]. Applying beneficial microbes in agriculture has a long history started from 60 years ago and becomes more supported as they were proven to reduce the biotic and abiotic stresses in plants [10]. Microorganisms that exist naturally in the soil are vital component of soil sub-ecosystem, since they play the key role in nutrient availability, reducing soil erosion and upgrading soil structure [11].

Mycorrhizae are associated with the majority of the plants under natural conditions [12]. Roots colonized by mycorrhizae are more efficient in nutrients acquisition, as its surface area can be extended up to several centimeters in soil [13]. Involvement of arbuscular mycorrhizal fungi(AMF) in micronutrient availability and mutualistic relationship construction with the roots have previously been deliberated [14]. The use of beneficial microbes and their products for agricultural purpose have many advantages such as bio-control agents without interrupting the ecological processes. On the other hand, the organism is chosen due to its resistance towards the specific chemical reagents. Furthermore, The self-replication of microbes may save the expenses of repeated applications [10, 15]. In addition to that, beneficial microbes can assist plants in transforming nutritional elements to available form and therefore holding a potential to ameliorate crop yields in an environmentally-friendlier manner [9, 16].

More than 90% of the plants establish a mutualistic relationship with AMF [17–19]. Previous studies supported the idea that AMF increases the level of secondary metabolites to assist the plant in resisting biotic and abiotic stresses [20]. *Piriformospora indica*, an axenically cultivable phytopromotional, biotrophic mutualistic root endosymbiont belongs to order Sebacinales (Basidiomycota). This fungus has a broad host range, which is not only confined to vascular plants but also to colonized mosses, implies that this fungus has evolved highly effective colonization strategies and provide plants multifaceted amenities (such as nutrient uptake, disease resistance, stress tolerance and growth- promotion involving value addition) [21–23]. In present study, the exogenous application of beneficial fungus *Piriformospora indica* on growth indices, phytochemical and health-promoting properties of Pakchoi was investigated by greenhouse experiments.

## Materials and methods

### Chemicals and reagents

Standard laboratory grade chemicals/reagents such as Folin-Ciocalteau reagent, 2, 2-diphenyl-1-picrylhydrazyl radical (DPPH), gallic acid, ascorbic acid, and (±)-6-Hydroxy-2, 5, 7, 8-tetramethylchromane-2-carboxylic acid (Trolox) were purchased from Sigma-Aldrich (USA). The phosphoric acid and DL-lactic acid were provided by Sangon Biotechnology; Biotin from Sinopharm chemical reagent Co., Ltd; whereas DTT (DL-Dithiothreitol), Trizma buffer, Nicotinamide (C_6_H_6_N_2_O), and Riboflavin (vitamin B2, C_17_H_20_N_4_O_6_) were procured from Shanghai Linfeng chemical reagent Co., Ltd. All other chemicals and solvents used were of analytical grade and used without any further purification.

### Greenhouse experiments

In the greenhouse experiment, the following 9 treatments were applied: (1) CK (un-inoculated sterile soil), (2) CF (chemical fertilizer), (3) OF (organic fertilizer), (4) B (biochar), (5) OB (organic fertilizer and Biochar), (6) BP (biochar and fungi), (8) OP (organic fertilizer and fungi), (9) OBP (organic fertilizer, biochar and fungi). The soil collected from an organic farm which located in Shanghai, China (30°51′ N 121°30′E) was air-dried, grounded, and passed through a 2.0 mm sieve. Soil basic properties were determined as; pH, 7.32; Electrical conductivity (EC), 0.14 (dS/m); available nitrogen, 111.6 (ppm); available phosphate, 181.7 (ppm), available potassium, 306.8 (ppm), cation exchange capacity (CEC), 13.2 (cmol(+)/kg); NH_4_^+^, 7.86 (ppm); NO_3_^-^, 2.67 (ppm); total carbon, 1.92 (%); total nitrogen, 0.19 (%); and total potassium, 2063 (ppm).

### Fertilizers and inoculation of soil

In treatment setup, analytical grade chemical fertilizers (urea and phosphorus for N source, and KH_2_PO_4_ as K source) were given with the following ratio; 0.6 g CO (NH_2_)_2_, 0.27 g KH_2_PO_4_ (N-P_2_O_5_-K_2_O = 0.28–0.14–0.13 g/pot). Organic fertilizer was prepared from chicken manure and mushroom waste and fermented for about 3 months with the following basic properties such as pH 7.9, water content 12.7%, OM 77.3%, total N 2.32%, P_2_O_5_ 4.51%, and K_2_O 2.56% (23.5 g per pot). Whereas, the Biochar provided by Seek Biotechnology Co., Ltd. Shanghai, China was prepared through the pyrolysis process of bamboo material under 400–500°C for 24 h and then filtered through 2.0 mm sieve (35 g per pot). The complete treatment setup is summarized in Table 1.

**Table 1:** Design of pot experiment used in this study.

Treatments	1	2	3	4	5	6	7	8	9
Mycorrhizal fungi	None	None	None	None	None	Yes	Yes	Yes	Yes
Biochar	None	None	None	Yes	Yes	Yes	None	Yes	None
Fertilizer used	None	CF	OF	None	O.F	None	OF	OF	None
code	C	CF	OF	B	OB	BP	OP	OBP	P

C Control. CF Chemical fertilizer. OF organic fertilizer. B Biochar. OB Organic fertilizer + Biochar. BP Biochar + Fungi. OP Organic fertilizer + Fungi. OBP Organic fertilizer + Biochar + Fungi. P Fungi

### Fungal inoculum preparation

The fungus *Pirimformospora indica* (CBS 125645) was obtained from “Centraal bureau voorSchimmel cultures, Fungal Biodiversity Centre, Institute of the Royal Netherlands Academy of Arts and Sciences (KNAW)”. It was inoculated on sterile petri plates containing kaefer medium and followed by incubation at 28°C for 6 to 8 days. For inoculation of soil substrate, the fungus was propagated for 15 days in Erlenmeyer flasks containing liquid kaefer medium under shaking conditions (120 rpm) to obtain a dense culture of mycelia.

### Plant material and growth conditions

Pakchoi seeds were procured from ShouguangRenhe Seed Industry Co., Ltd. China. Uniform and healthy seeds were surface sterilized using 10% peroxide solution for 30 min [24] and then germinated in petri plates on sterilized wet filter paper. Two-day-old vigorous and healthy seedlings were transplanted to pots (top diameter 14 cm, bottom diameter 10 cm, height 12 cm) and sandwich layer model was applied to inoculate the soil with *P*. *indica* [25, 26]. Pots were randomly placed in green house at an optimum temperature of 15–17°C. Three plants per pot were grown with replication numbers of 12 for each treatment and harvested after 45 days.

### Total RNA extraction and cDNA synthesis

Total RNA was extracted from fresh leaves samples collected from six weeks old pakchoi plants using TaKaRa plant Mini kit according to the manufacture’s guidelines. The concentration of RNA was measured using a Nanodrop-2000 spectrophotometer (Thermo Scientific, USA), furthermore, the quality was confirmed by agarose gel electrophoresis. Prior to reverse transcription reaction, RNA samples were treated with DNase (TaKaRa, Japan) to remove genomic DNA. RNA was transcribed into cDNA using TaKaRa Reverse Transcription kit according to manufacture’s instructions (TaKaRa, Japan). The synthesized cDNA was quantified by a spectrophotometer (Eppendorf, Germany) at 260 nm.

### Quantitative real-time PCR analysis

**Fig 1:**
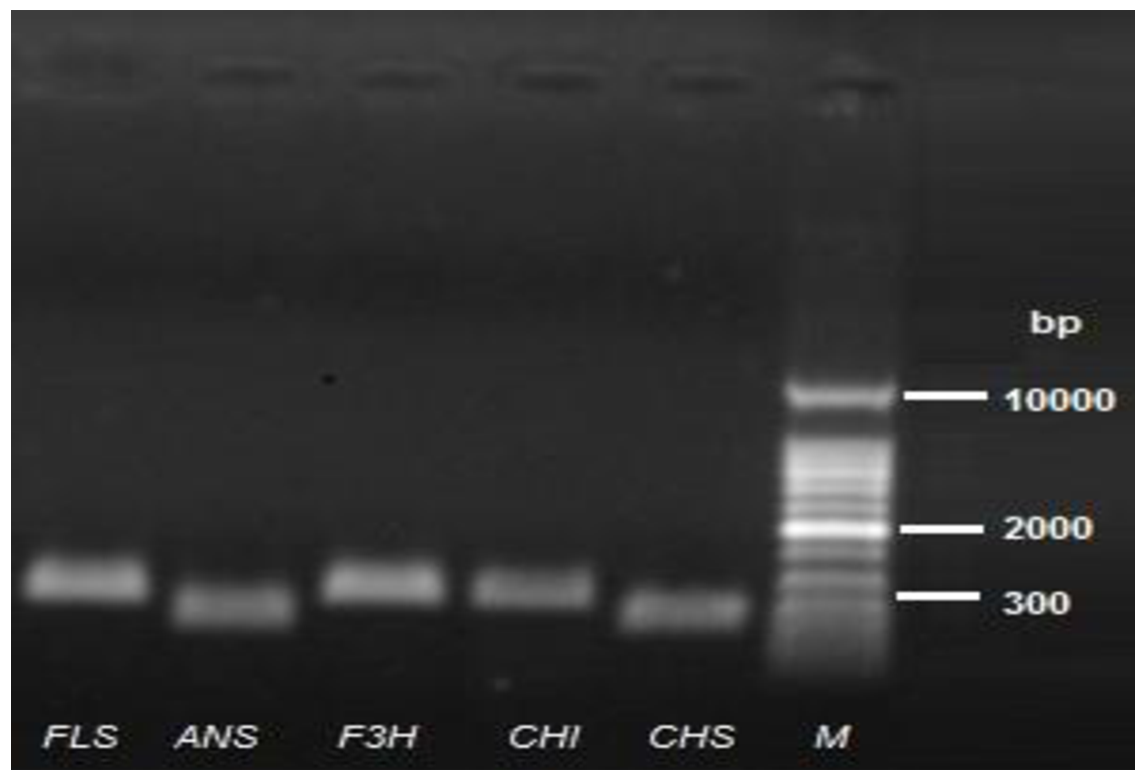
Electrophoresis profiles of the flavonoid biosynthesis pathway genes.

**Fig 2:**
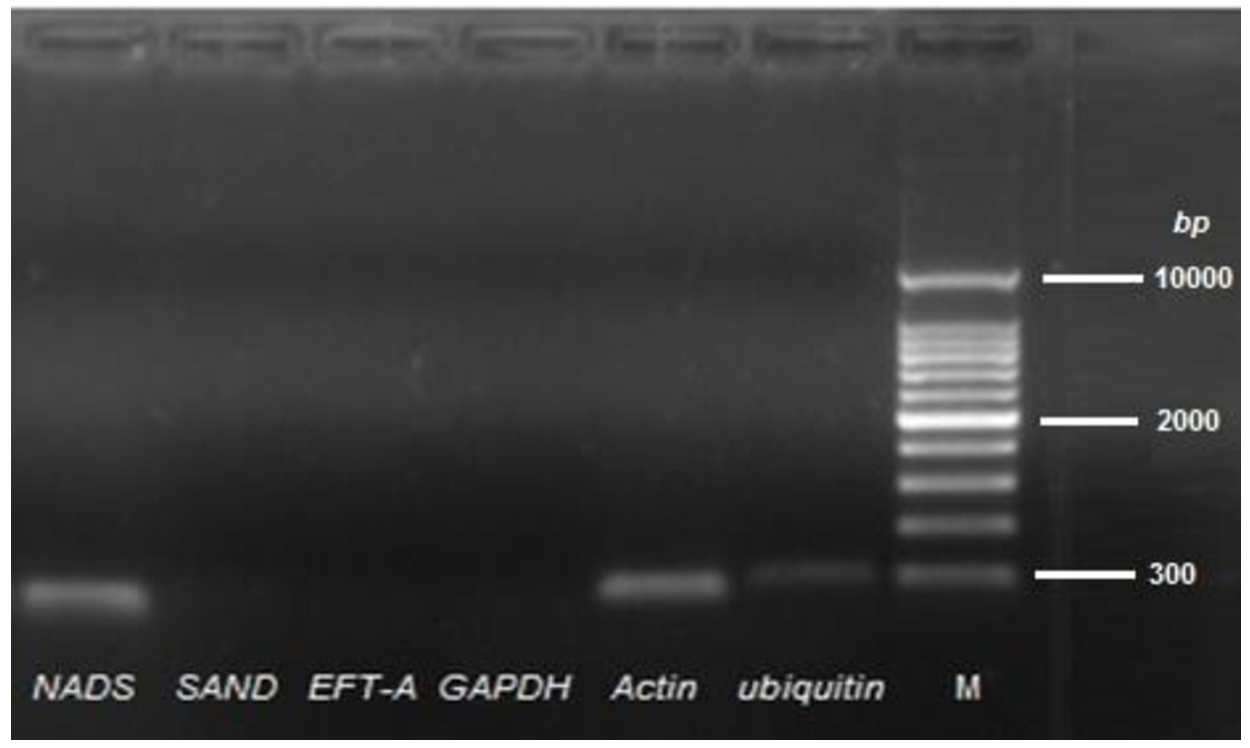
Electrophoresis profile of candidates for housekeeping genes.

The expression levels of flavonoid biosynthetic pathway genes from Pakchoi were assessed by quantitative real-time PCR (qRT-PCR) using a Light Cycler^®^ real-time PCR system (96 version 1.1.0-.1320, Roche Diagnostics international Ltd.) with SYBR^®^ Premix Ex Taq^™^(TaKaRa, Japan). Two sets of gene-specific primers were designed for each gene (Table 2) by using GenScript Real-time PCR (TaqMan) online Primer Design tool (https://www.genscript.com/ssl-bin/app/primer). Referred to the relevant studies [22], five genes were selected and tested to be used as the internal control (Table 3). The qRT-PCR was performed in three independent experimental repeats using a 20 μl total volume, where each experimental repeat contains at least three samples. The assembly of reaction mixture and amplification were carried out as previously described by Hassani et al. [27]. The best gene-specific primers and internal control for qRT-PCR were selected based on their electrophoresis profiling (Figs 1 and 2). Calculation of relative expression level was performed by 2^–ΔΔCT^ method [28].

**Table 2:** Primers designed for qRT-PCR analysis of flavonoid biosynthesis genes in *Brassica campestris ssp*. *chinensis L*.

Gene	Forward primer (from 5’ to 3’)	Reverse primer (from 5’ to 3’)	T_m_(°C)
*CHS*	**CATCTGACACCCACCTTGAC**	GAAGATGGGCTTCTCTCCAG	58.5
	GAGAGAAGCCCATCTTCGAG	**GTCCCACTTCCCTCAAGTGT**	57.2
*CHI*	**TCCCTTTCTTCCGTGAAATC**	TTCCATATCGCCACACAGTT	54.7
	AACTGTGTGGCGATATGGAA	**GAGAGCGAAGAGGATGGAAG**	55.7
*F3H*	**GTCCCAAGGTTGCCTACAAT**	**CCAGTTCTCACAAGCCTCAA**	56.1
	ATAGCCACGTTCCAGAATCC	CTCCAAGATCGGCTTCTCTC	57.0
*ANS*	GGGATCAGCTCTATCCCAAA	GGACTTGTGGACCGTCTTCT	56.5
	**GGTTTGCAGCTGTTCTACGA**	**CACCAATCCACGGTGAAGTA**	55.2
*FLS*	**AATTACTATCCGCCGTGTCC**	**TTGACGTCGATCCAGTGATT**	55.5
	ACTTCCGGAATCATCGTCAT	AAACCGGCCATGATATTCTC	56.2

Note: The primers which were finally selected for qRT-PCR analysis were shown in bold font.

**Table 3:** List of candidate housekeeping genes primers.

Gene ID	Forward primer	Reverse primer
Ubiquitin	TCTGAGGCTTCGTGGTGGTA	AGGCGTGCATAACATTTGCG
Actin*	**CTTGCATCCCTCAGCACCTT**	**TCCTGTGGACAATGGATGGA**
GAPDH	TTCTCGTTGAGGGCTATTCCA	CCACAGACTTCATCGGTGACA
Eft-a	GAACTGGGTGCTTGATAGGC	AACCAAAATATCCGGAGTAAAAGA
SAND	CAACATCCTTTACCCATTGACAGA	GCATTTGATCCACTTGCAGATAAG
NADS	GATGCTTCTTGGGGCTTCTTGTT	CTCCAGTCACCAACATTGGCATAA

*The selected primer has been bolded.

### Growth and root analysis

Growth attributes such as leaf number, leaf area, fresh and dry biomass were determined after harvesting the experimental samples under different treatments. The root colonization was assayed by using trypan blue staining kit [(protein and cell biology) life science products and services, Sangon Biotech (shanghai) Co., Ltd.] following the method of Smith et al. [29] and Newman’s intersection method with minor modifications [30].

### Measurement of total phenolic compounds

Folin–Ciocalteau method was used to determine the total phenolic compounds (TPC) in sample extract [31]. Briefly, 500 μl of experimental sample in water was mixed with 2.0 ml of Folin–Ciocalteau reagent (0.2 N). After three min, 10 ml of Na_2_CO_3_ (10%, w/v) was added and the resulting mixture was allowed to stand for 30 min in the dark. The absorbance was measured at 725 nm against a blank and the results were expressed as mg gallic acid per gram of fresh weight of the sample.

### Measurement of total flavonoid content

Analysis of total flavonoid content was carried out following the method as reported earlier [32]. One ml of aluminum chloride (2.0%, *w/v*) was thoroughly mixed with 1.0 ml of crude sample, followed by incubation at room temperature for 10 min. Results of total flavonoids were calculated by measuring optical density at 430 nm and expressed in mg quercetin per gram of fresh weight of the sample.

### Measurement of phenolic acid content

A previously reported method was adopted for measuring the phenolic acid contents (PACs) in the extracted sample [33]. To this end, 1.0 mL of sample was thoroughly mixed with a combination of 5.0 mL of sterilized distilled water, 1.0 mL HCl (0.5 M), Arnov reagent (100 ml H_2_O, 10 g sodium nitrite and 10 g sodium molybdate) and NaOH (1.0 M) followed by OD measurement at 590 nm. A calibration curve was constructed to measure the total PAC_S_ and the results were presented equivalent as caffeic acid in micro gram per gram of fresh weight of the sample.

### HPLC-MS analysis

Quantitative assessment of phenolic compounds was carried out through HPLC-MS (LTQ XL, Thermo Fisher Scientific, San Jose, CA, USA) using a C18 column (2.1 mm×150 mm, 3.5 μm; Waters) [34]. The column temperature was maintained at 35°C. The mobile phase A (0.1% formic acid/water) and B (100% acetonitrile) was used; the gradient program was as follows: 0–2 min 5.0% B; 4–11 min 15%-35% B; 15–17 min, 100% B; 17.5–22 min, 5.0% B; flow rate was 0.30 ml min^-1^, the injection volume was 10 μl. MS was scanned in ESI source in negative mode, mass range: *m/z* 92 to 1000; source voltage 3.5 kV, capillary temperature 350°C, sheath gas flow 35, aux gas flow 15.0, sweep gas flow 1.0, and a capillaryvoltage of 43V. The dependent scan was performed with collision-induced dissociation (CID) at collision energy of 35 eV. Data acquisition, handling, and instrument control was performed using X calibur 2.3.1 software.

### Chlorophylls and carotenoids measurement

Chlorophyll and carotenoid levels were evaluated as reported [35, 36]. Briefly, a 2.0 ml acetone (80%) was used overnight at 4°C to elute chlorophyll and carotenoids from 0.05 g freeze-dried leaves. Supernatants were collected after centrifugation of the sample at 13,000 rpm for 5.0 min. The absorbance was recorded at wavelengths of 663, 645, and 470 nm for chl a, chl b, and carotenoids, respectively and concentrations of chl a, chl b and carotenoids were measured using the equations given below:

$$\text{Chlorophyll a }=\;12.72\times {\text{OD}}_{663}-2.59\times {\text{OD}}_{645}$$

$$\text{Chlorophyll b }=\;22.88\times {\text{OD}}_{645}-4.67\times {\text{OD}}_{663}$$

$$\text{Carotenoids }=\;(1000\times {\text{OD}}_{470}-3.27\times \text{chla}-104\times \text{chlb})÷229$$

### Total antioxidant activity

Free radical scavenging assay was carried out to determine antioxidant activity using 1, 1-diphenyl-2-picrylhydrazyl (DPPH) as reported earlier [37]. An 80 μl of methanolic sample extract was mixed with 1.92 ml DPPH solution, and absorbance was noted at 515 nm.

### Statistical analysis of data

All the analytic determinations were carried out at least in three times, and results are expressed as mean ± *SD* of triplicate samples. Data were statistically analyzed by one-way analysis of variance (ANOVA), followed by Duncan’s multiple range (DMR) tests (SPSS Inc., Chicago, IL, USA). Differences were denoted statistically significant at *P*<0.05.

## Results and discussion

### Morphological indices

**Fig 3:**
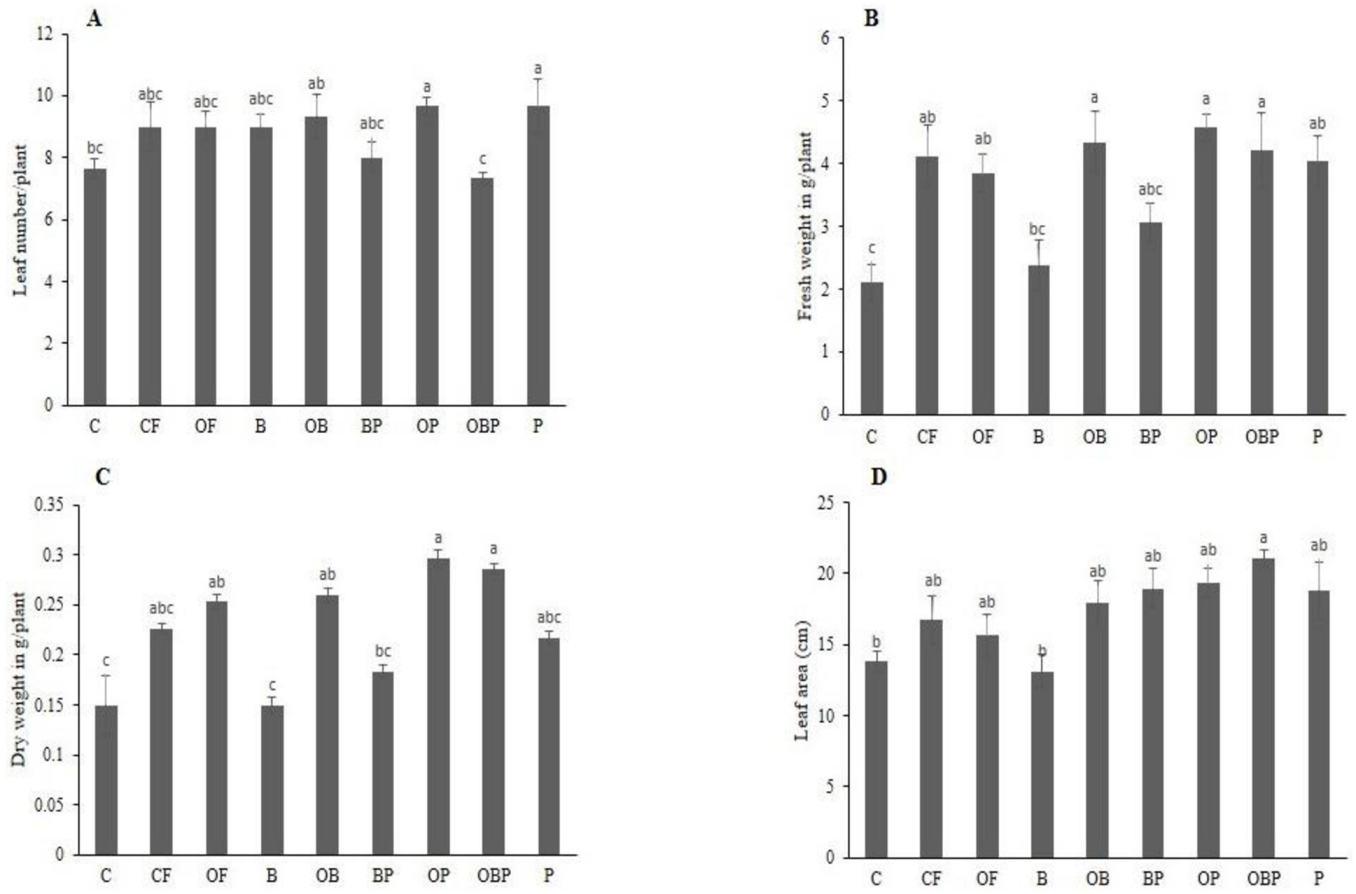
Effect of given treatments on growth of *Brassica campestris* ssp. *chinensis* L. A) Leaf number, B) Shoot fresh weight, C) Shoot dry weight, D) Leaf area. Values are means and bars indicate SDs (*n* = 8). Columns with different letters indicate significant difference at *P* < 0.05 (Duncan test). Treatments Control (C), Chemical fertilizer (CF), organic fertilizer (OF), Biochar (B), Organic fertilizer + Biochar (OB), Biochar + Fungi (BP), Organic fertilizer + Fungi (OP), Organic fertilizer + Biochar + Fungi (OBP), Fungi (P).

The morphological indices of crop Pakchoi were evaluated following nine selected treatments and results are illustrated in (Fig 3A–3D). The plant characteristics such as leaf number, fresh weight, dry weight and leaf area were significantly (*P*<0.05) varied between the treatments. Notably, the leaf numbers were found higher with treatment P (55.8%) and lowest in treatment OBP. Fresh and dry weights were increased up to 68.18% and 65.9% respectively in OP compared to control treatment. The leaf area was maximum in OBP (60.48%) and minimum in B (48.77%) treatments. In summary, the results showed that *P*. *indica*-inoculated plants exhibited excellent growth as compared to control and other treatments (S1 Fig).

Similar findings representing the growth enhancing features of *P*. *indica* inoculation have been documented earlier [25, 38, 39]. A considerable increase in the fresh weight of seedlings was recorded in *P*. *indica* inoculated *Arabidopsis* plants [40]. Similarly, the *P*. *inidica* treated *C*. *forskohlii* resulted a substantial increase in biomass including aerial growth, leaf area and the average length of the branches [41] which might be due to the higher expression of genes responsible for development [42]. Moreover, the growth-promoting effects could also be associated with high nutrient uptake especially nitrogen and phosphorus from the soil [43, 44]. Several researchers demonstrated that *P*. *indica* assists in phosphorus uptake and involves in phosphorus shipping through PiPT transporter [45–47].

### Roots colonization assay

**Fig 4:**
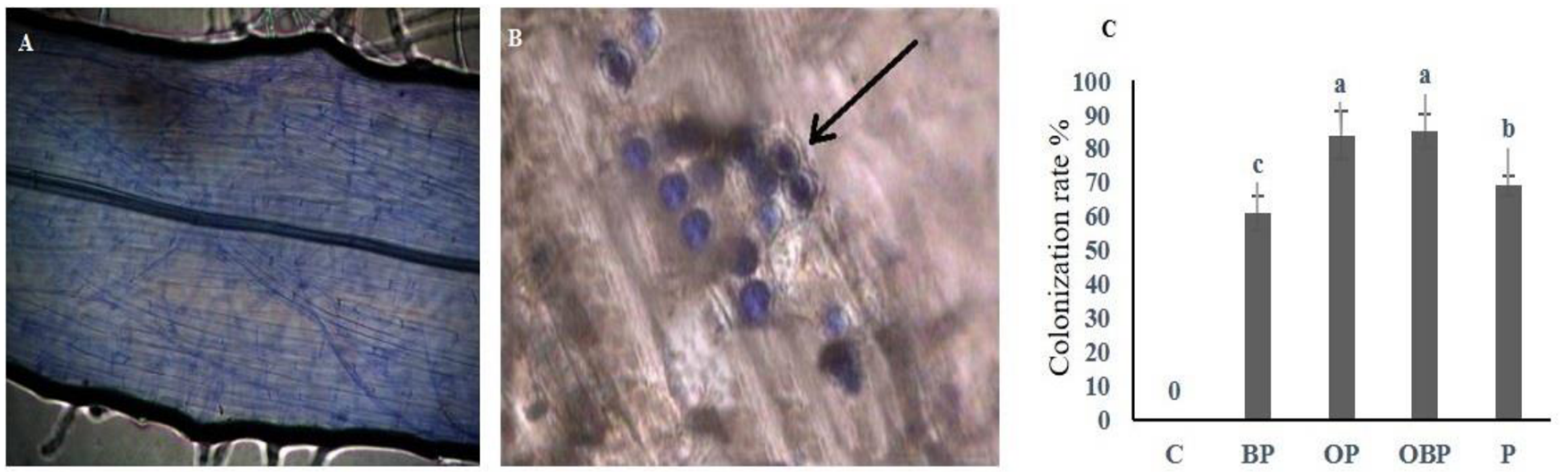
Root colonization and sporulation of *P*. *indica* in *Brassica campestris* ssp. *chinensis* L., A) Control, B) Chlamydospores inside the root cells, C) Plant root infection rate by *P*. *indica* in different treatments. Alphabets on bars significantly differ at p <0.05. Treatments Control (C), Biochar + Fungi (BP), Organic fertilizer + Fungi (OP), Organic fertilizer + Biochar + Fungi (OBP), Fungi (P).

Roots colonization in treated plants was monitored under a microscope. Colonization in the form of mycelia, hyphae, and mature piriform shaped chlamydo spores was observed in primary and secondary roots (Fig 4). The use of staining technique thus confirmed the presence of beneficial endophytic fungus inside the inoculated sample roots (Fig 4). Comparable results were observed previously with *Arabidopsis thaliana*, *Zea mays*, *Hordeumvulgare*, *Oryza sativa* and many other monocots and dicots etc. [29].

### Health-promoting compounds analysis

**Fig 5:**
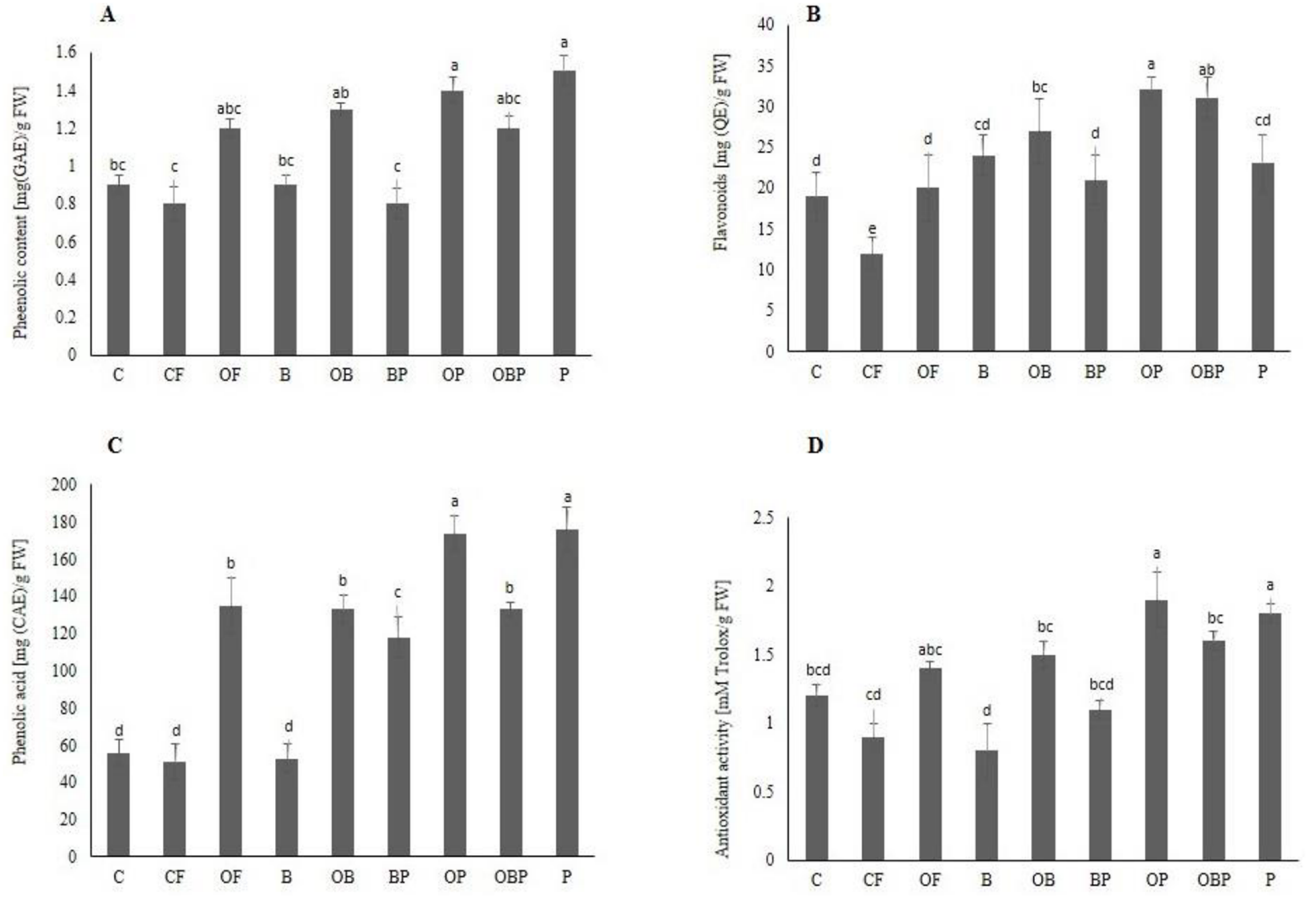
Influence of different treatments on A) phenolic contents as gallic acid equivalent (GAE) in mg per g of fresh weight (FW) B) Flavonoids as Quercetin equivalent (QE) in μg per g of fresh weight (FW)., C) phenolic acid as caffeic acid equivalent (CAE) in μg per g of fresh weight (FW)., D) Antioxidant activity. Treatments Control (C), Chemical fertilizer (CF), organic fertilizer (OF), Biochar (B), Organic fertilizer + Biochar (OB), Biochar + Fungi (BP), Organic fertilizer + Fungi (OP), Organic fertilizer + Biochar + Fungi (OBP), Fungi (P).

In order to determine the nutritional quality of Pakchoi, it is of profound significance to investigate the content and the activity of health-promoting phytochemicals. Thus, in the present study, the quality of the Pakchoi was determined by analyzing the concentration of different secondary metabolites especially antioxidants (total phenolics, flavonoids, and phenolic acid) under given treatments. It was observed that phytochemicals were significantly augmented in fungal-inoculated plant samples in combination with organic fertilizer and biochar in comparison to control and other treatments (Fig 5A–5D). It is worth to mention that the elevated synthesis of phenolic content, as well as flavonoids, correspond perfectly with the results obtained by Kilam et al. [48]. Treatments such as OB (59.09%), OP (60.86%) and P (62.5%) presented the most promising effects on the plant phenolic contents. The concentration of flavonoids increased considerably as a consequence of OBP (62%) and OP (62.7%) treatments as compared to control and other treatments (Fig 5A–5D). Likewise, plants treated with OP (75.6%) and P (75.86%) accumulated higher concentrations of phenolic acid. Analogous effects have been observed for some other plants such as *Hordeumvulgare*, *Stevia rebaudiana*, *Oryza sativa* L., and *Bacopamonniera* [48–51].

Antioxidants exhibit strong curative or preventive activities by inhibiting many cellular pathways which are crucial for chronic ailments such as neurodegenerative, cardiovascular diseases and cancer [52–54]. Key pathways such as inflammatory, detoxification, immune response, cell division and proliferation, growth and differentiation are regulated by the action of specific enzymes that can be induced or inhibited by flavonoids. A vast array of biological functions have been attributed to flavonoids since it can modify immune system, influence cancer at any stage and homeostasis in cell system [55, 56]. The antioxidant capacity in *Brassica* species might be ascribed to the presence of flavonoids and phenolic contents as compared to vitamins and carotenoids [57]. This has been demonstrated that Pakchoi is rich in beneficial phytochemicals that are correlated with environmental biotic and abiotic factors [34].

The possible compounds were identified by HPLC-MS and compared with the reported literatures about *Brassica campestris* ssp. *chinensis* L. (S2 and S3 Figs). The retention time, *m/z* in negative mode, MS^2^ fragments, and the possible chemical name are detailed in Table 4. Results showed that the dominant fractions in Pakchoi were found to be Ferulic acid, Caffeic acid, Kaempferol and Luteolin. Noticeably, one of the most important flavonoids “Quercetin” was not detected in control and under chemical fertilizer treatment while it was present in OP associated plants (Table 4). Additionally, the quantities of Caffeoyltartaric acid, Phillyrin, Isorhamnetin-3-Gentiobioside-7-glucoside, Kaempferol, Chlorogenic acid, Caffeic acid and Ferulic acid were found to be higher in OBP and followed by OP inoculated plants. Moreover, the level of Ferulic acid was increased at high degree with OP, OBP, and P, similarly, the Caffeic acid enhancement was evident in plants elicited with OP, B and P treatments. Previous studies also supported that kaempferol, isorhamnetin. quercetin, flavonoid derivatives and other imperative polyphenols were relatively higher in different varieties of Pakchoi following fungal inoculation [58, 59]. [60]. An improved biosynthesis of phenolic content and flavonoids might be related to the symbiosis which involves a molecular dialogue between beneficial fungus and plant [61, 62]. Such an induction of potentially beneficial compounds in lettuce associated with fungi has also previously been reported [17]. In another study, an elevated level of phenolic compounds (chlorogenic acid, gallic acid, Hydroxy benzoic acid) was recorded in *Valerianajatamansi Jones* when treated with mycorrhiza as compared to non-inoculated one’s [63].

**Table 4:** Quantitative analysis of phenolic compounds in the leaves of *Brassica campestris ssp*. *chinensis L*. after treatments.

μg/mL	C	CF	OF	B	OB	BP	OP	OBP	P
Ferulic acid	0.66 ± 0.02 cde	0.54 ± 0.01 de	0.57 ± 0.15 de	0.73 ± 0.007 abc	0.65 ± 0.50 bcd	0.48 ± 0.8 e	0.83 ± 0.10 a	0.8 ± 0.13 ab	0.74 ± 0.05 abc
Caffeic acid	0.7 ± 0.05 i	2.68 ± 0.39 h	4.87 ± 0.19 g	7.42 ± 0.50 b	6.63 ± 0.32 d	6.44 ± 0.009 e	9.23 ± 0.001 a	5.81 ± 0.22 f	7.1 ± 0.01 c
Chlorogenic acid	3.45 ± 0.09 a	4.85 ± 0.10 a	4.26 ± 0.12 a	4.09 ± 0.76 a	6.3 ± 0.39 a	5.66 ± 0.17 a	6.21 ± 0.05 a	5.69 ± 0.005 a	6.45 ± 0.10 a
Kaempferol	0.21 ± 0.15 e	0.32 ± 0.67 d	0.41 ± 0.05 c	0.22 ± 0.39 e	0.42 ± 0.40 c	0.48 ± 0.25 b	0.68 ± 0.67 a	0.67 ± 0.02 a	0.53 ± 0.33 b
Luteolin	0.02 ± 0.004 d	ND	0.05 ± 0.39 cd	0.02 ± 0.04 d	ND	0.08 ± 0.005 ab	0.09 ± 0.04 a	0.07 ± 0.05 abc	0.06 ± 0.44 bc
Caffeoylmalic acid	8.81 ± 0.5 b	10.69 ± 0.05 ab	10.95 ± 0.18 ab	11.73 ± 0.25 ab	10.31 ± 0.08 ab	10.74 ± 0.03 ab	12.22 ± 0.44 ab	13.96 ± 0.16 ab	11.08 ± 0.16 ab
Coumaric acid	0.19 ± 0.13 d	0.18 ± 0.005 d	ND	0.26 ± 0.44 bc	0.18 ± 0.16 d	0.21 ± 0.44 cd	0.31 ± 0.007 ab	0.34 ± 0.25 a	0.26 ± 0.54 bc
Quercetin-3-gentiobioside-7-glucoside	ND	0.03 ± 0.02 e	0.08 ± 0.31 cd	0.1 ± 0.14 cd	0.1 ± 0.04 cd	0.06 ± 0.16 de	0.28 ± 0.52 a	0.15 ± 0.03 b	0.11 ± 0.22 bc
Isorhamnetin-3-Gentiobioside-7-glucoside	0.13 ± 0.03 de	0.13 ± 0.18 de	0.08 ± 0.67 e	0.2 ± 0.06 d	0.27 ± 0.03 bc	0.22 ± 0.007 c	0.33 ± 0.13 b	0.43 ± 0.02 a	0.26 ± 0.005 c
Quercetin	ND	ND	0.02 ± 0.18 d	0.03 ± 0.22 d	0.14 ± 0.01 c	0.18 ± 0.05 b	0.25 ± 0.008 a	0.24 ± 0.14 a	0.19 ± 0.02 b
5-*p*-coumaroylquinic acid	6.24 ± 0.17 ab	4.77 ± 0.76 b	4.46 ± 0.001 b	6.62 ± 0.05 ab	8.01 ± 0.005 a	6.84 ± 0.01 ab	9 ± 0.11 a	6.77 ± 0.10 ab	9.41 ± 1.59 a
Schisantherin D	0.29 ± 0.20 a	ND	0.17 ± 0.42 cd	0.27 ± 0.18 ab	0.25 ± 0.18 abc	0.24 ± 0.32 abc	0.2 ± 0.42 bc	0.28 ± 0.42 ab	0.12 ± 0.05 d
Phillyrin	0.12 ± 0.07 e	0.16 ± 0.02 de	0.18 ± 0.03 bc	0.24 ± 0.01 a	0.24 ± 0.33 a	0.12 ± 0.02 e	0.22 ± 0.22 ab	0.26 ± 0.05 a	0.17 ± 0.15 bcd
Caffeoyltartaric acid	0.58 ± 0.43 c	0.73 ± 0.13 bc	0.72 ± 0.04	0.63 ± 0.005 bc	0.8 ± 0.15 b	0.76 ± 0.005 bc	1.01 ± 0.03 d	1.28 ± 0.03 a	0.8 ± 0.03 b

Treatments Control (C), Chemical fertilizer (CF), organic fertilizer (OF), Biochar (B), Organic fertilizer + Biochar (OB), Biochar + Fungi (BP), Organic fertilizer + Fungi (OP), Organic fertilizer + Biochar + Fungi (OBP), Fungi (P). Values are means and bars indicate SDs. Columns with different letters indicate significant difference at P < 0.05 (Duncan test).

#### Chlorophylls and carotenoids

Like other green leafy vegetables, pakchoi is a persuasive source of dietary chlorophylls and carotenoids that play a striking role in reducing the risk of heart disease, cancer, cataract, stroke and in-vitro anti-inflammatory effects [64, 65]. Carotenoids are also secondary metabolites with profound antioxidant activities, whereas some studies revealed that chlorophyll causes the inhibition of Cox-1 and Cox-2 enzymes [65, 66]. The results in Table 5 showed that chlorophyll contents were markedly increased as a result of CF and OP treatments followed by P and OBP treatments as compared to control, however, no significant difference (*P*>0.05) was found between control and all other treatments. Chlorophyll b contents were higher with P and OP treatments followed by OB treatment in comparison to control and other treatments. Likewise, chlorophyll a, b were recorded highest in chemical fertilizer and OP treatments. While there was no significant difference (*P*>0.05) among other treatments, The results were in consonance with previous investigations that *P*. *indica* possess beneficial influence on chlorophyll content and photosynthetic efficiency [67, 68]. Some reports also showed that *P*. *indica* confers stress resistance and enhance fresh weight and chlorophyll content in the model plant, *Arabidopsis thaliana*. Data regarding carotenoids evaluation showed that Pakchoi treated with OP was categorized to have significantly higher (*P*<0.05) total carotenoids followed by B and OBP treatment with respect to other treatments. Improvement of carotenoid content in the *P*. *indica* associated plants has previously been explicated in several studies [69, 70]. Similarly, biosynthesis of a higher level of carotenoids and photosynthetic pigments (chl a, b) have also been reported in *P*. *indica*-inoculated rice seedlings as compared to non-inoculated seedlings [71].

**Table 5:** Influence of given treatments on chlorophyll and carotenoids contents.

Treatment	Constituents (mg/100 g dw)			
	Chl a	Chl b	Chl a + b	Car
C	338.45±3.7 d	121.31 ± 21.17 de	459.76 ± 24.87 f	25.74 ± 0.8 c
C.F	473.22 ± 17.45 a	208.20 ± 8.60 a	681.42 ± 26.05 a	30.96 ± 1.76 bc
O.F	341.32 ± 7.62 cd	131.74 ± 2.42 bc	473.06 ± 10.04 e	18.45 ± 3.08 d
B	340.28 ± 13.40 cd	120.74 ± 2.42 e	461.02 ± 15.62 f	34.51 ± 1.54 b
OB	344.44 ± 17.22 cd	136.81 ± 11.03 b	481.25 ± 28.25 d	24.21 ± 1.24 cd
BP	348.81 ± 17.22 c	128.83 ± 6.25 cd	477.64 ± 23.47 de	27.32 ± 2.12 c
OP	468.25 ± 21.40 a	215.44 ± 17.08 a	683.69 ± 38.48 a	41.23 ± 2.32 7 a
OBP	434.71 ± 21.40 b	127.28 ± 13.40 cde	561.99 ± 34.8 c	34.51 ± 1.55 b
P	439.23 ± 22.44 b	214.62 ± 17.04 a	653.85 ± 39.48 b	29.32 ± 2.14 bc

Abbreviations: Chl a, chlorophyll a; Chl b, chlorophyll b; Car, carotenoids. Treatments Control (C), Chemical fertilizer (CF), organic fertilizer (OF), Biochar (B), Organic fertilizer + Biochar (OB), Biochar + Fungi (BP), Organic fertilizer + Fungi (OP), Organic fertilizer + Biochar + Fungi (OBP), Fungi (P). Values are means and bars indicate SDs. Columns with different letters indicate significant difference at P < 0.05 (Duncan test).

### Expression level of flavonoid pathway genes

**Fig 6:**
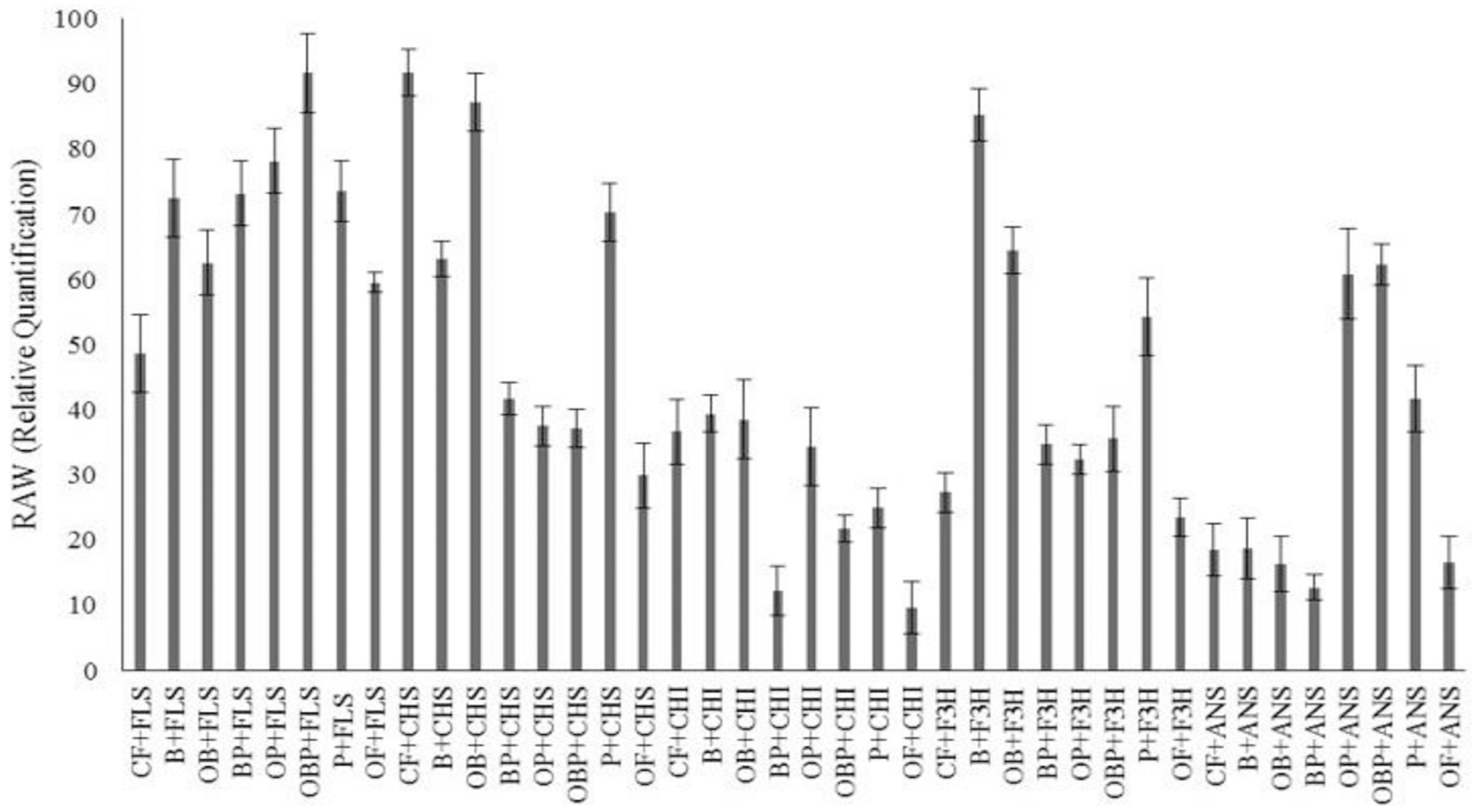
QRT-PCR analysis of the expression levels of the FLS, CHI, ANS, CHS and F3’H genes from flavonoids biosynthesis pathway.

To date, seventy-three anthocyanin biosynthetic pathway genes (ABGs) have been characterized in the genome of *Brassica campestris* ssp. *chinensis* L. Structural genes accompanying this pathway can be categorized into(1) early flavonoid biosynthesis genes, including *CHS*, *CHI* and *F3H*, and (2) late flavonoid biosynthesis genes, such as *FLS* and *ANS* [72]. In flavonoid biosynthetic pathway, the synthesis of flavonoids begins with the precursor, 4-coumaroyl-CoA. Enzymes encoded by the genes *CHS*, *CHI*, *F3H*, *FLS* and *ANS* convert this precursor to several intermediate compounds which are used as a substrate for the next step of the pathway. Kaempferol as a product of FLS is known to be the major flavonoid in *Brassica campestris* ssp. *chinensis* L. In this experiment, the expression level of five important structural genes of flavonoid biosynthetic pathway in pakchoi was determined and quantified using RT-qPCR in nine different treatments (Table 1). Results proposed that the expression level of early genes of the flavonoid pathway including *CHS*, *CHI* and *F3H* were considerably higher in treatment by Biochar followed by OB. On the other hand, the expression level of late flavonoid pathway genes including *FLS* and *ANS* was recorded to be significantly higher in OBP and OP treatments. The expression profile of individual genes under each treatment has been illustrated in Fig 6.

**Fig 7:**
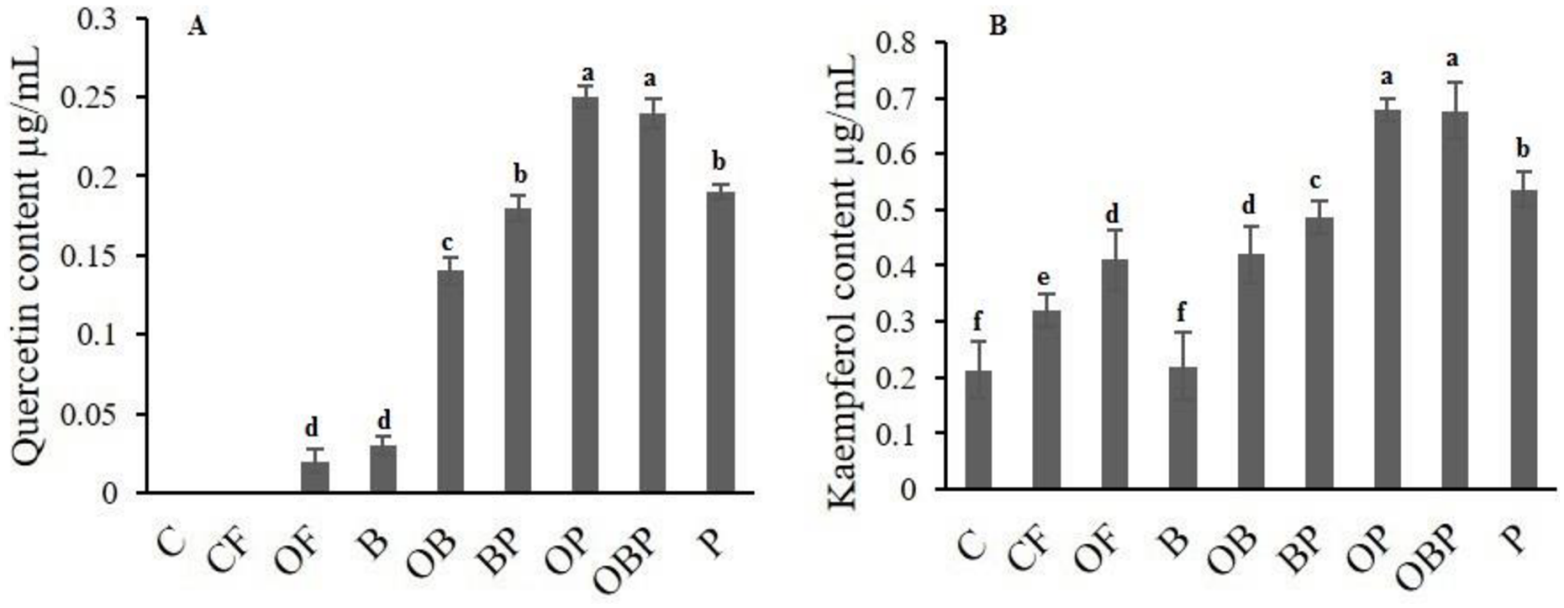
Major flavonoids detected by HPLC A) Quantitative assessment of quercetin and B) Kaemferol by HPLC. Treatments Control (C), Chemical fertilizer (CF), organic fertilizer (OF), Biochar (B), Organic fertilizer + Biochar (OB), Biochar + Fungi (BP), Organic fertilizer + Fungi (OP), Organic fertilizer + Biochar + Fungi (OBP), Fungi (P).

Data from qRT-PCR analysis revealed that OBP and OP significantly (*P*<0.05) affected the expression level of *FLS* and *ANS* which are involved in major flavonoids biosynthesis (Fig 7A and 7B). More importantly, the spectrophotometric results, HPLC and expression level of late genes in flavonoids biosynthesis pathway (*FLS*, *ANS*) coincided well and therefore, it is concluded that *P*. *indica* in combination with organic fertilizer and Biochar can induce the synthesis of flavonoids in the host plant. Similar results have been reported earlier [51, 73–75].

### Free radical scavenging activity

DPPH free-radical scavenging assay was used to determine the antioxidant activity which was increased by P, OP, OBP and OB treated crop as compared to control and other treatments. The highest capacity reached to 1.9 and 1.8 mM of TE/g FW for OP and P treated which constitute up to 61.29% and 60%, respectively in comparison to control. The higher antioxidant capacity might be attributed to the flavonoids and phenolic contents, which were significantly influenced by fungi whose positive effect was evident from the above-mentioned data (Table 4, Fig 5A–5D). Besides antioxidant properties, phenolic compounds also possess the anti-inflammatory activity by inhibiting enzymes involved in inflammation process [65, 76]. Previous reports elaborated that *B*. *monniera* has shown a drastic increase in antioxidant activity when treated with *P*. *indica*. The level of major antioxidants (i.e., flavonols and caffeic acid derivatives) can be amplified by AMF in lettuce plant when studied comparatively to the non-inoculated plants [17]. Extrapolating all the results achieved after supplying nine different treatments along with previous results, it is summarized that overall positive change could be achieved in Pakcoi by intervention with *P*. *indica*.

## Conclusions

In conclusion, the results showed that OP and OBP treatments have a favorable effect on the concentration of beneficial nutrients and therefore can improve the nutritional quality of pakchoi. In addition to that, the elevated levels of phenolics, flavonoids, and phenolic acid were found in co-cultivated plants with *P*. *indica* compared to non-inoculated ones. These findings reveal that treatment with beneficial fungus as bio-fertilizer can be a cost-effective and environmentally-friendlier approach for enhancing the quality and health properties of fresh vegetables, which may act as an alternative to conventional chemical-based strategies. In-depth studies on unraveling the contribution of beneficial fungi and its relationship with different host plants would be important for future sustainable agriculture

## Supporting information

S1 Fig The appearance of Pakchoi plant cultivated under given treatments. (DOCX)

S2 Fig The HPLC-UV chromatogram of sample [(A) 370 nm, (B) 270nm]. (DOCX)

S3 Fig Mass spectra of identified compounds. (DOCX)

S4 Fig Amplification profile of target genes using SYBR green. (DOCX)

## Abbreviations

B: Biochar
BP: Biochar + Fungi
C: Control
CF: Chemical fertilizer
chla: chlorophyll a
chlab: chlorophyll ab
Chlb: chlorophyll b
DW: dry weight
FW: fresh weight
OB: Organic fertilizer + Biochar
OBP: Organic fertilizer + Biochar + Fungi
OF: organic fertilizer
OP: Organic fertilizer + Fungi
P: Fungi
