# Supplementary material for: Elevation of secondary metabolites synthesis in Brassica campestris ssp. chinensis L. via exogenous inoculation of Piriformospora indica with appropriate fertilizer
Source: PLoS One. 2017 May 11;12(5):e0177185. doi: 10.1371/journal.pone.0177185 (PMC5426706; doi:10.1371/journal.pone.0177185)
Supplement: S1 Fig — (DOCX) [file pone.0177185.s001.docx]

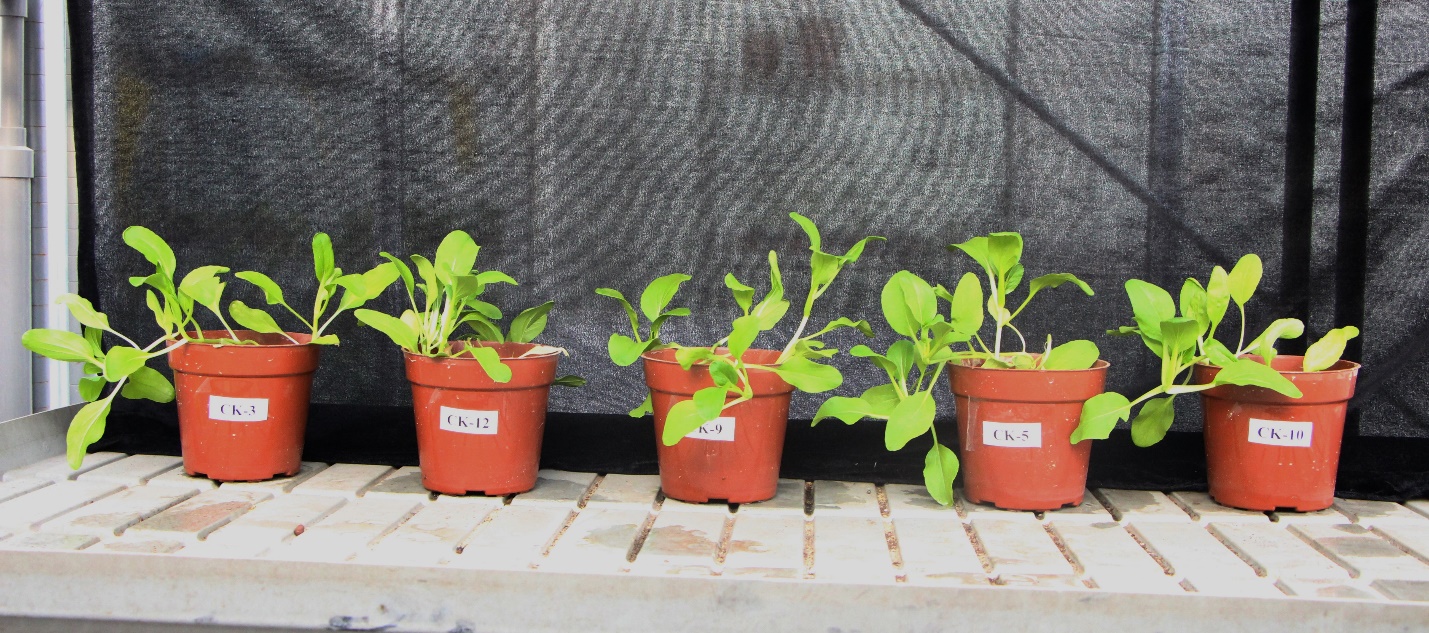


Control (C)


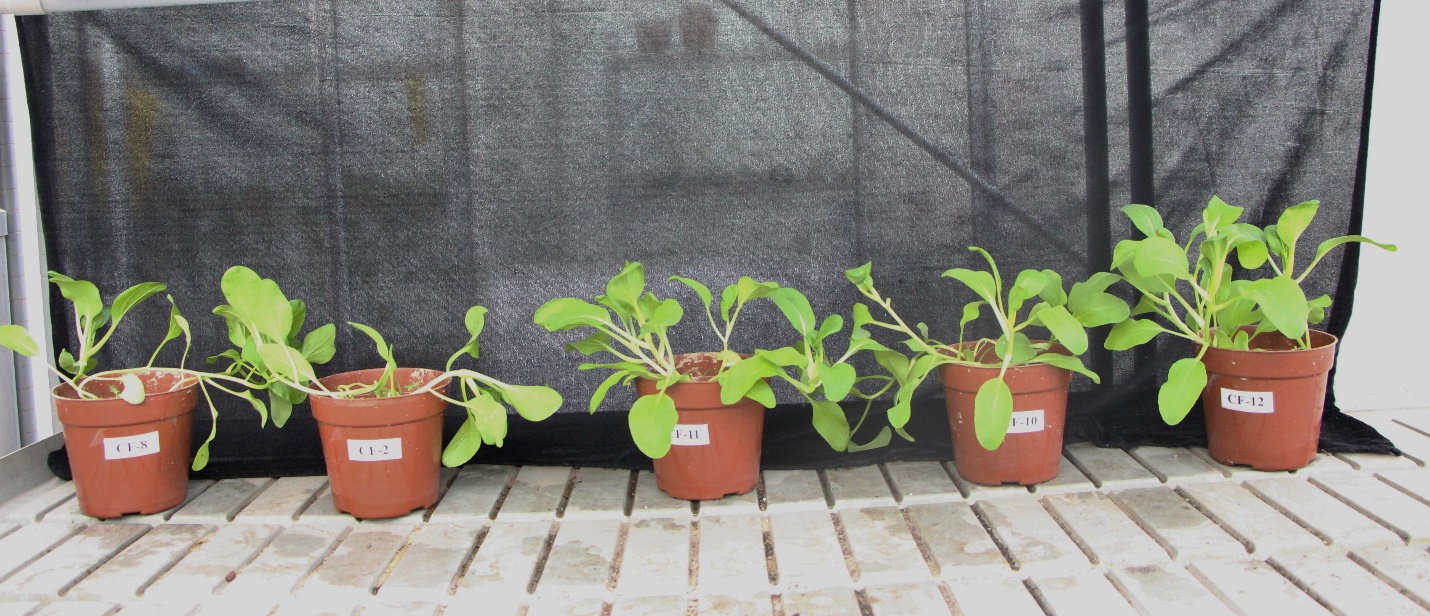


Chemical fertilizer (CF)


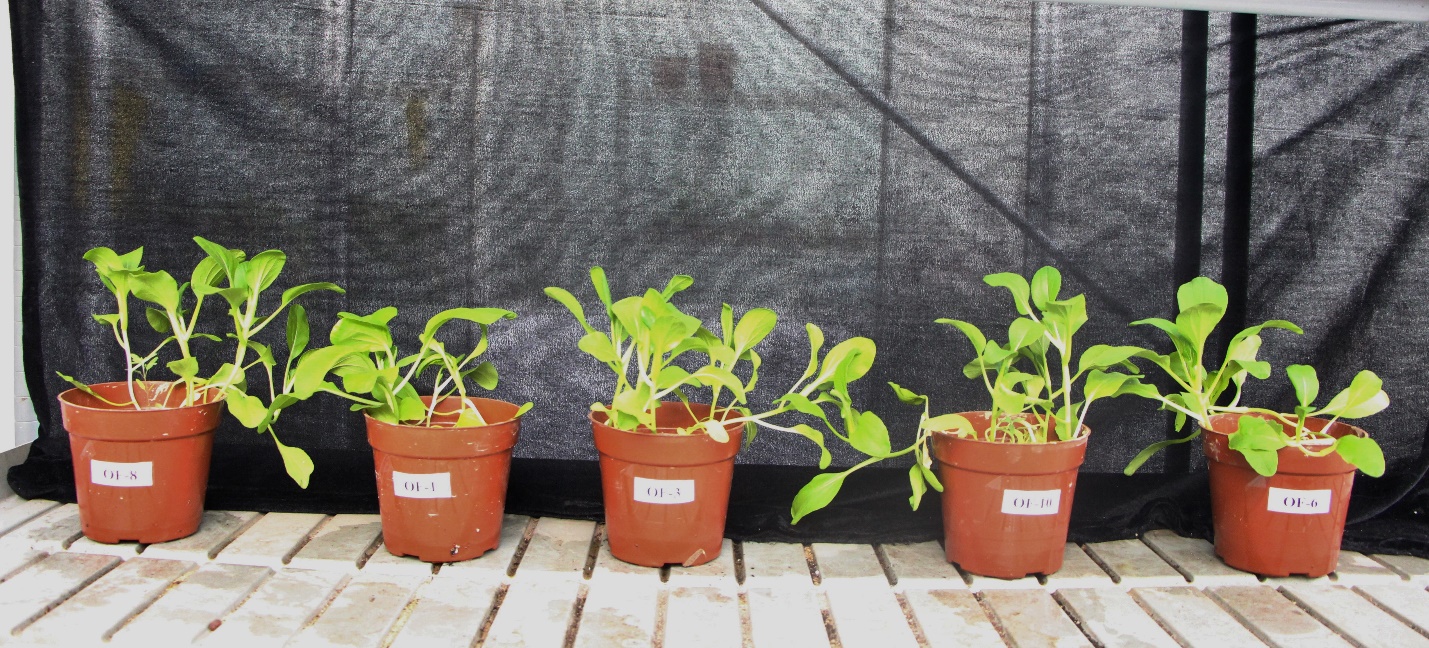


Organic fertilizer (OF)


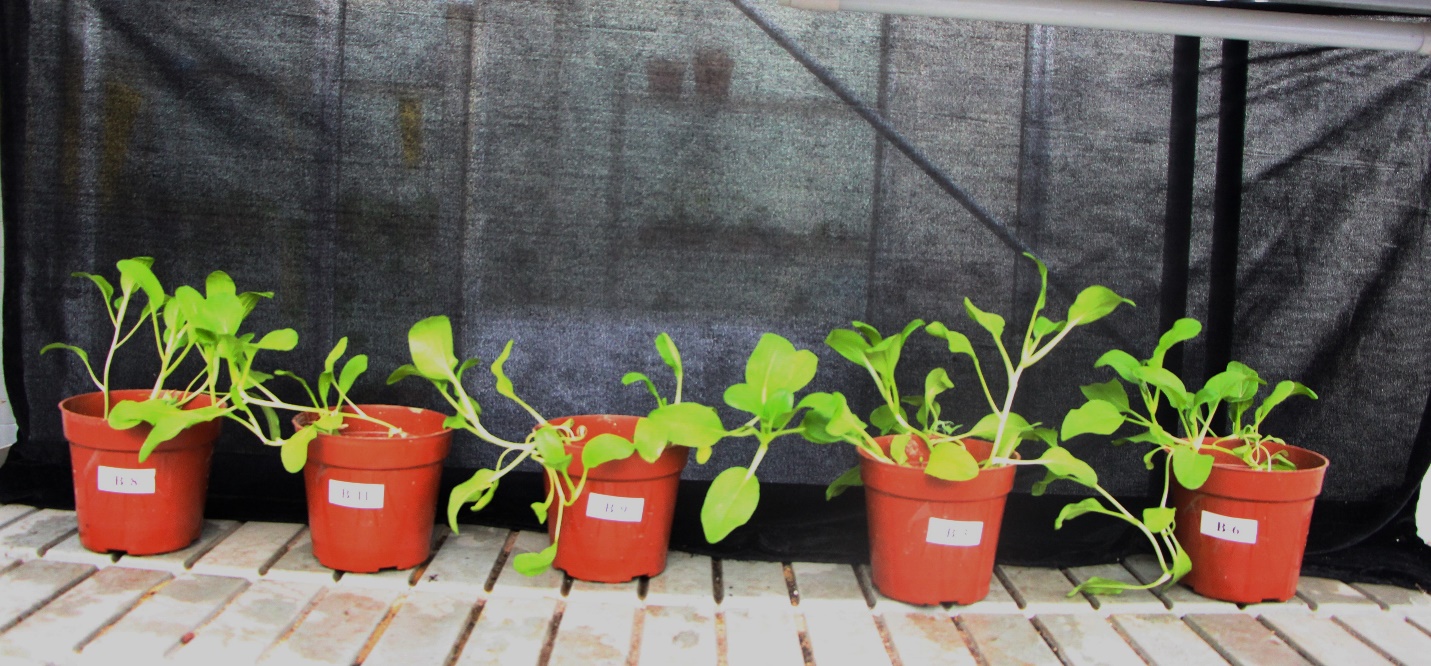


Biochar (B)


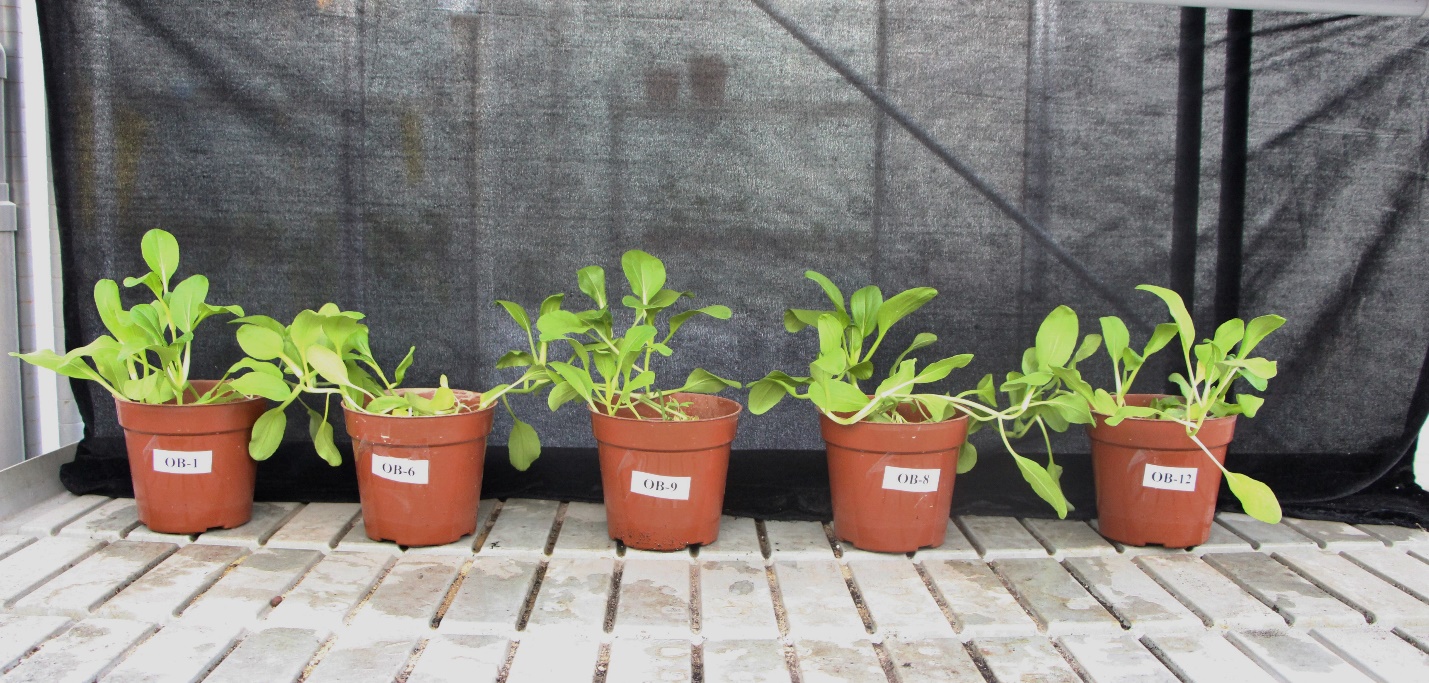


Organic fertilizer + Biochar (OB)


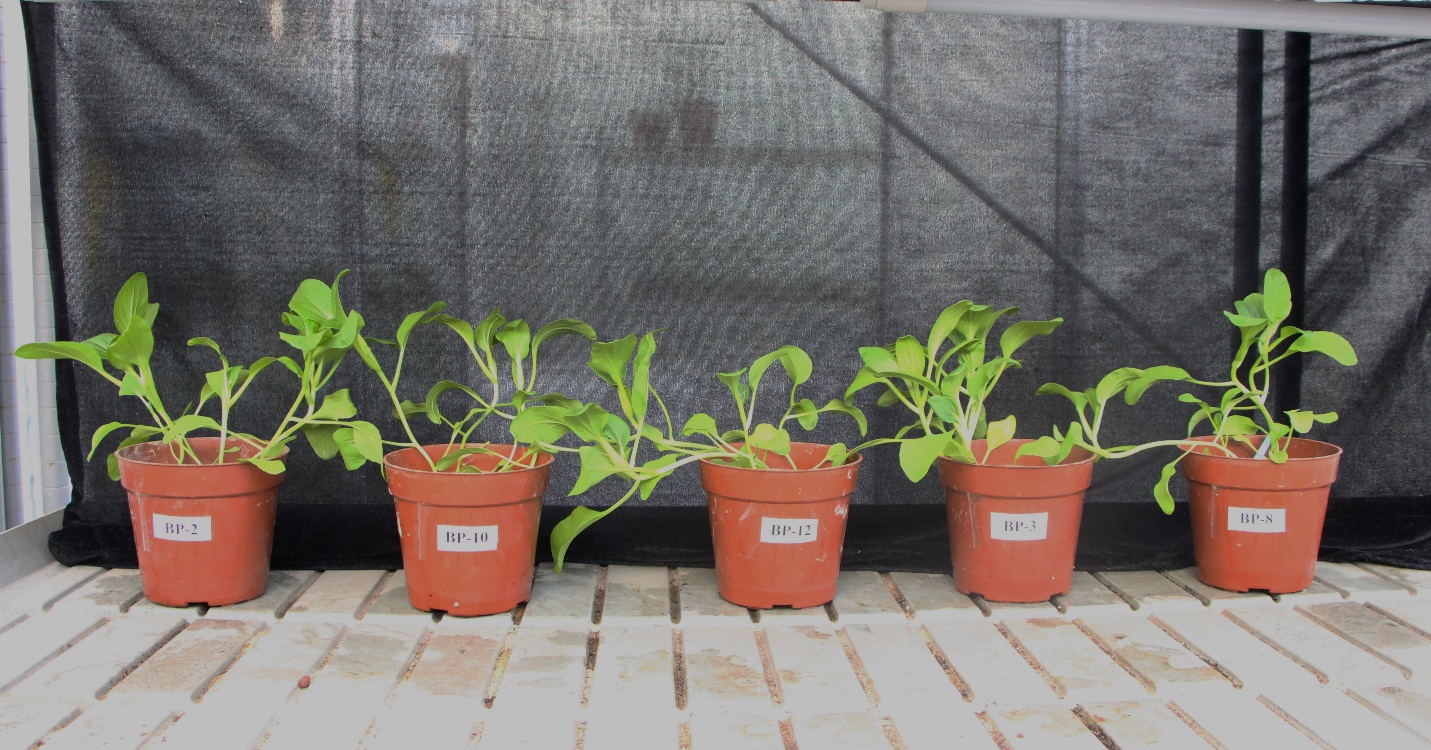


Biochar + Fungi (BP)


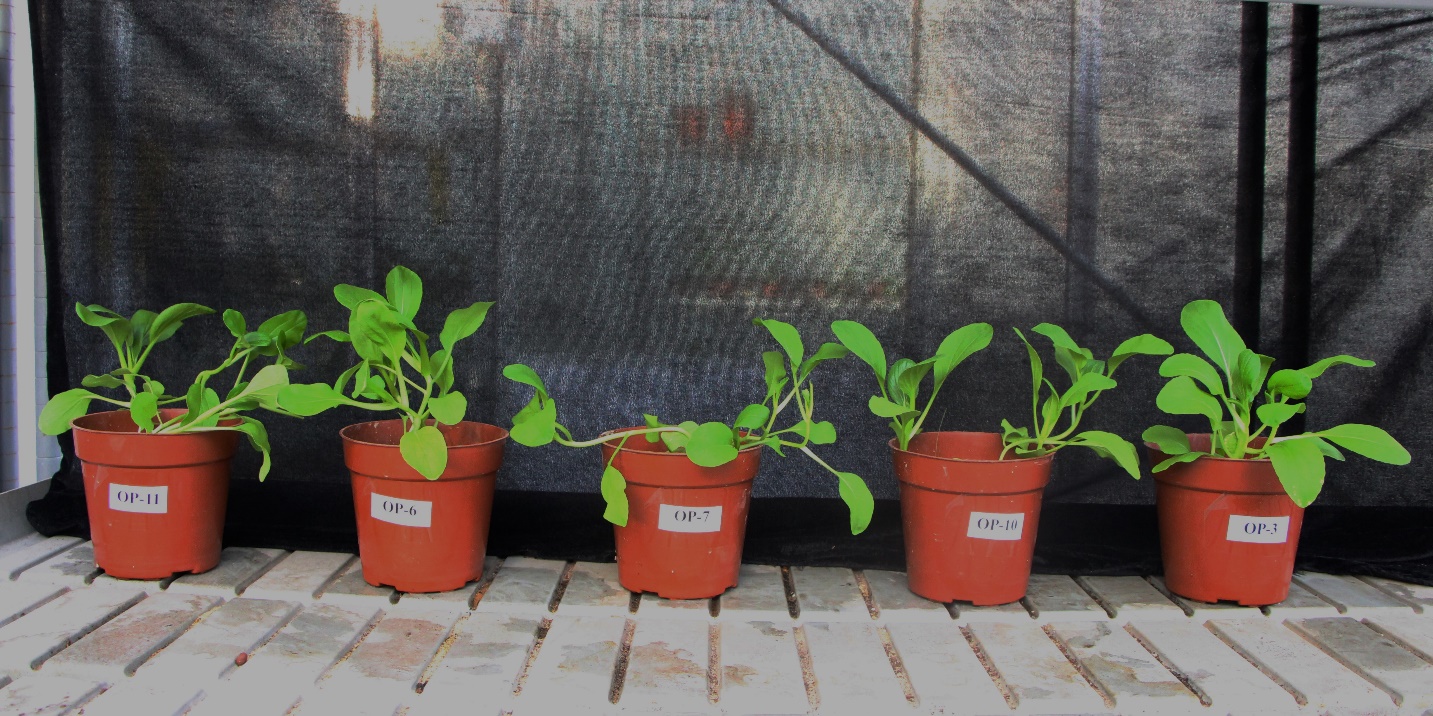


Organic fertilizer + Fungi (OP)


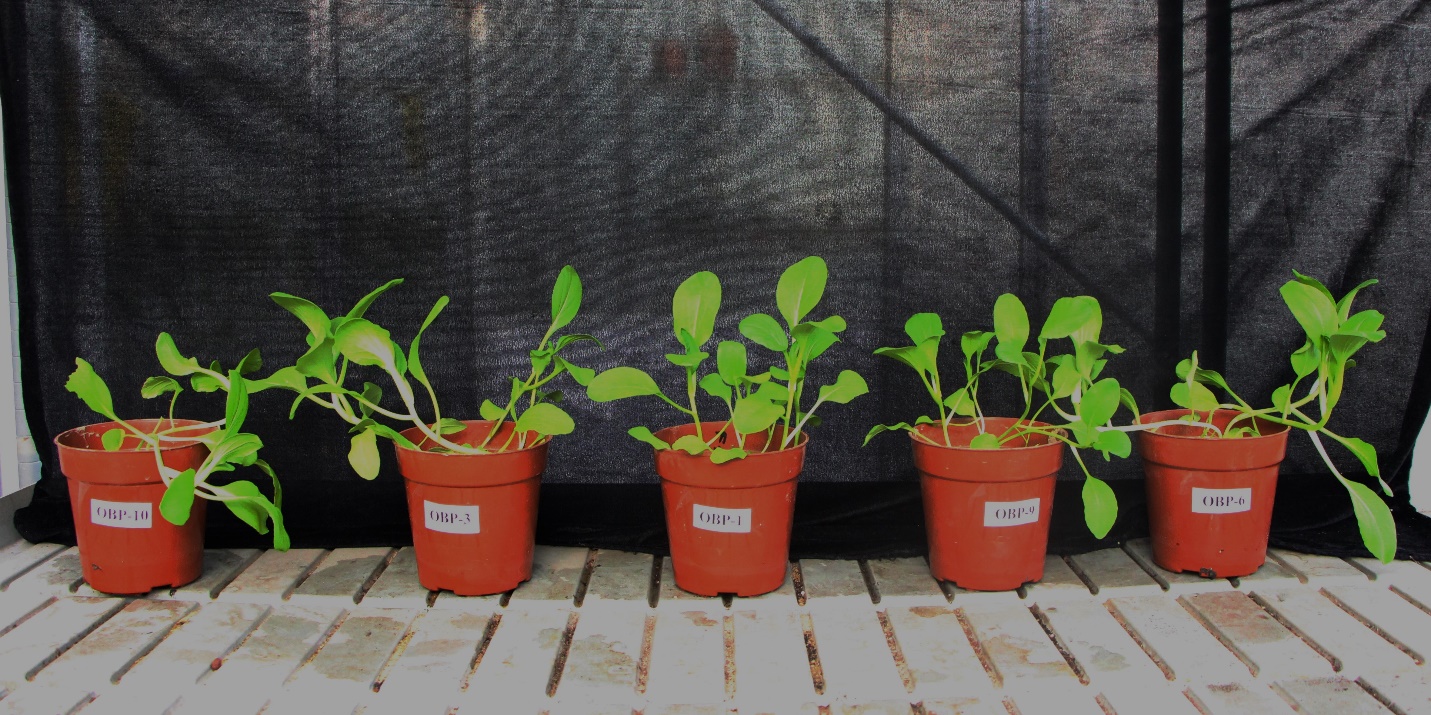


Organic fertilizer + Biochar + Fungi (OBP)


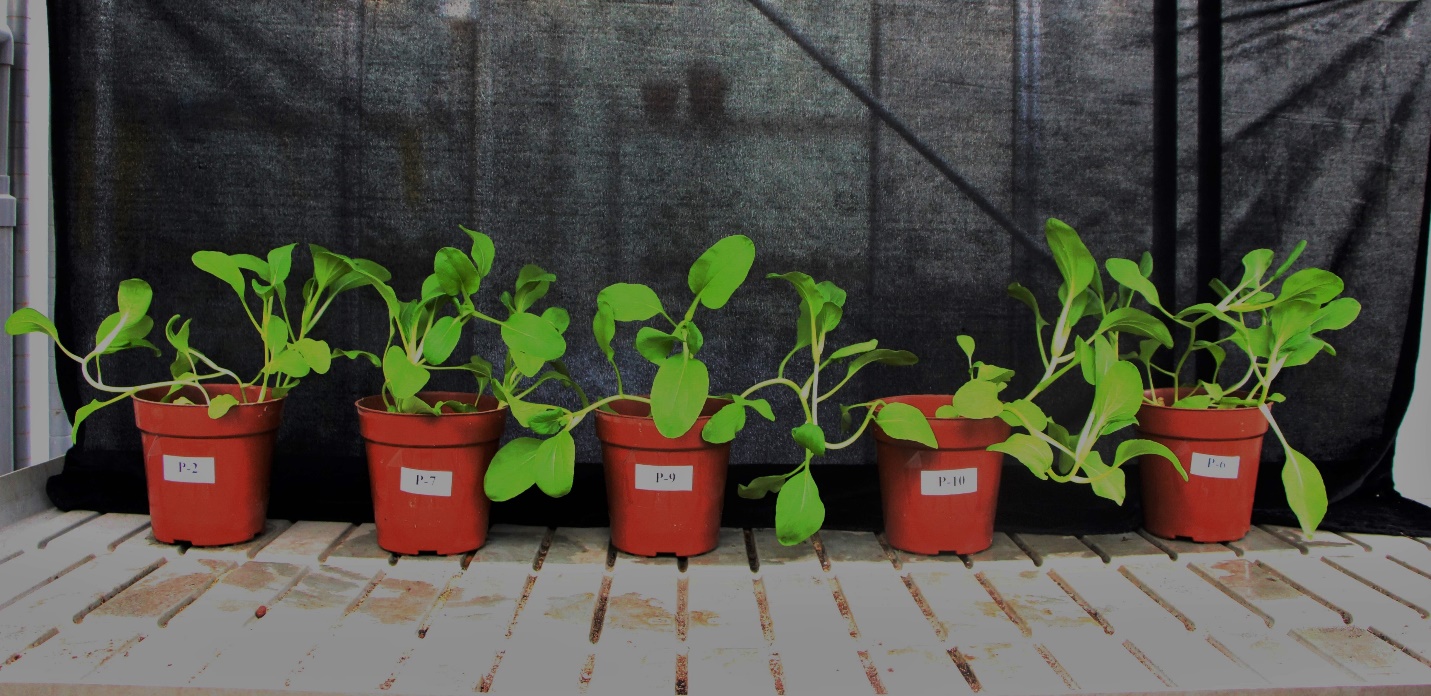


Fungi (P)

S1 Fig: The appearance of Pakchoi plant cultivated under given treatments.
