# Supplementary figures and images for: Elevation of secondary metabolites synthesis in Brassica campestris ssp. chinensis L. via exogenous inoculation of Piriformospora indica with appropriate fertilizer
Source: PLoS One. 2017 May 11;12(5):e0177185. doi: 10.1371/journal.pone.0177185 (PMC5426706; doi:10.1371/journal.pone.0177185)

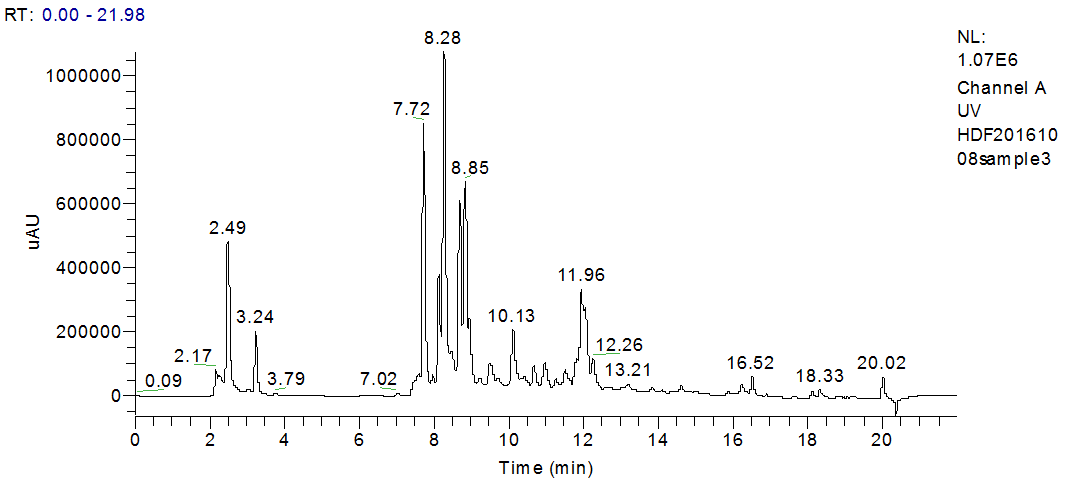


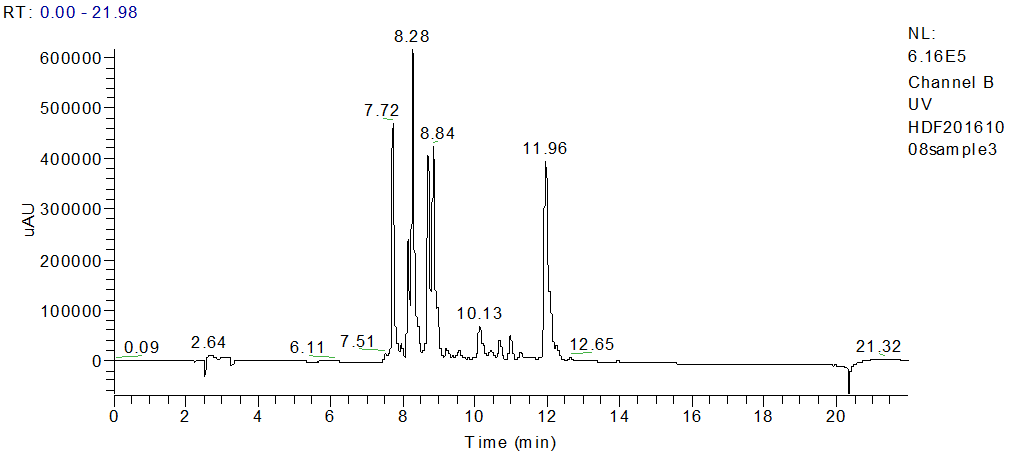


S2 Fig: The HPLC-UV chromatogram of sample [(A) 370 nm, (B) 270nm].

Supplement: S2 Fig — (DOCX) [file pone.0177185.s002.docx]

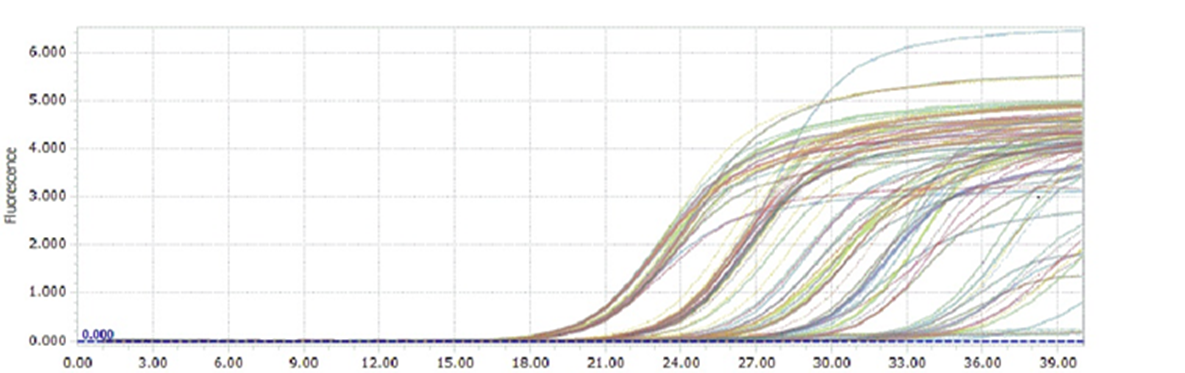


S4 Fig: Amplification profile of target genes using SYBR green

Supplement: S4 Fig — (DOCX) [file pone.0177185.s004.docx]
