# Supplementary material for: Elevation of secondary metabolites synthesis in Brassica campestris ssp. chinensis L. via exogenous inoculation of Piriformospora indica with appropriate fertilizer
Source: PLoS One. 2017 May 11;12(5):e0177185. doi: 10.1371/journal.pone.0177185 (PMC5426706; doi:10.1371/journal.pone.0177185)
Supplement: S3 Fig — (DOCX) [file pone.0177185.s003.docx]

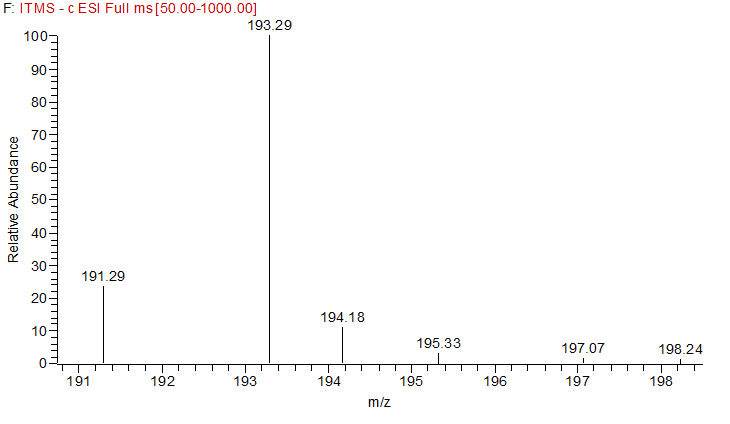


The mass spectrum of ferulic acid


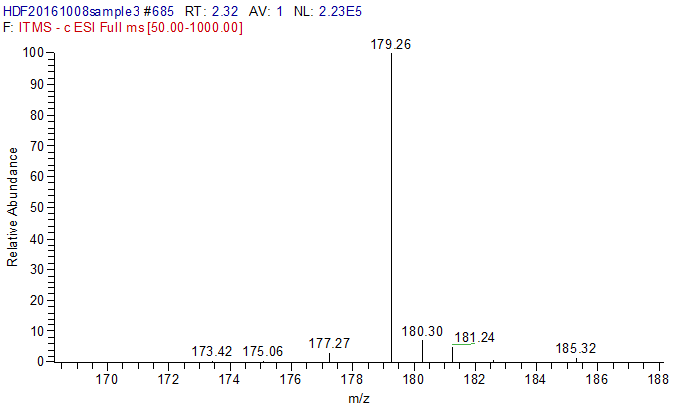


The mass spectrum of caffeic acid


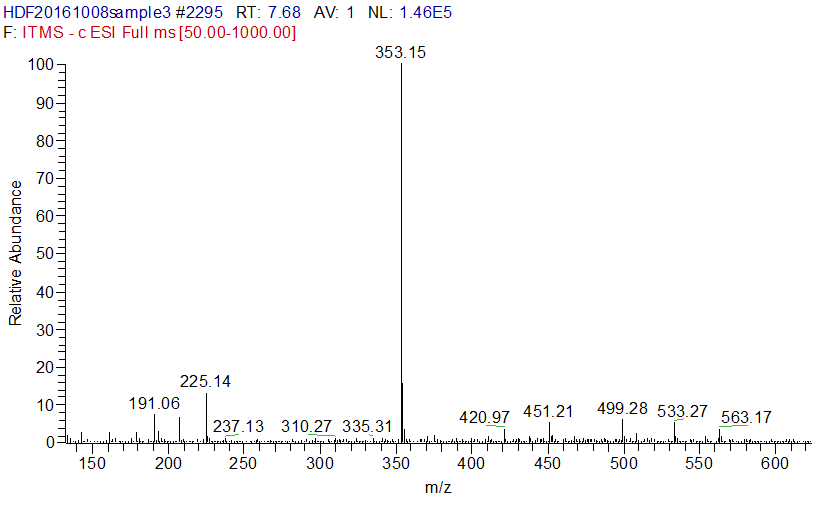


The mass spectrum of chlorogenic acid


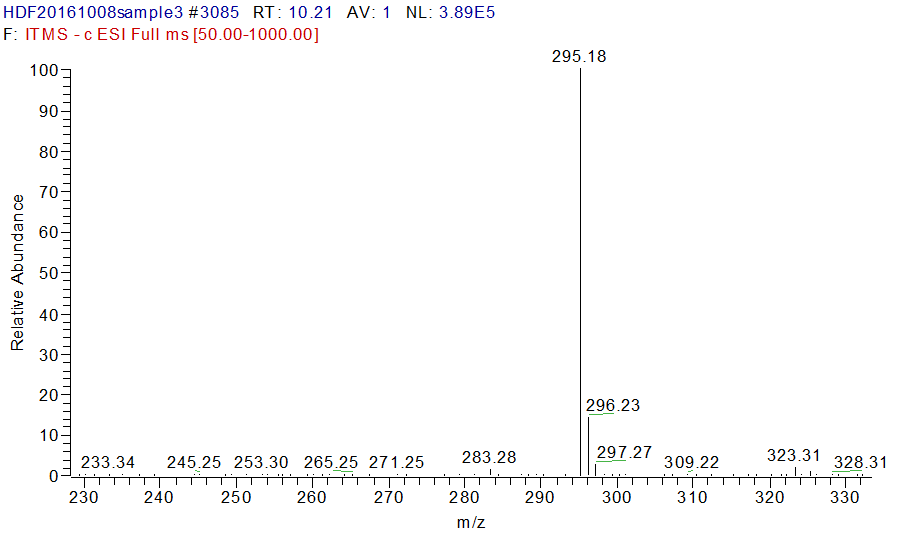


The mass spectrum of caffeoylmalic acid


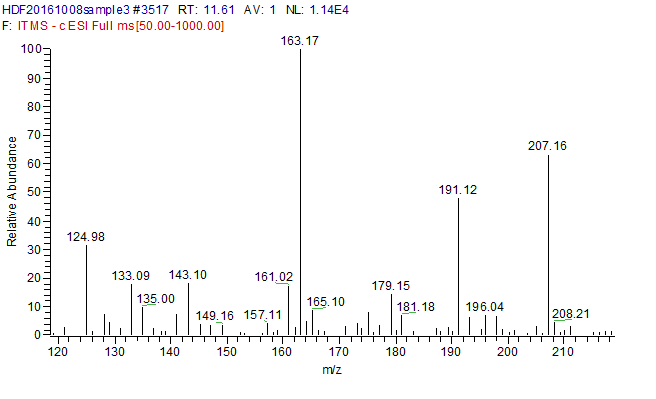


The mass spectrum of coumaric acid


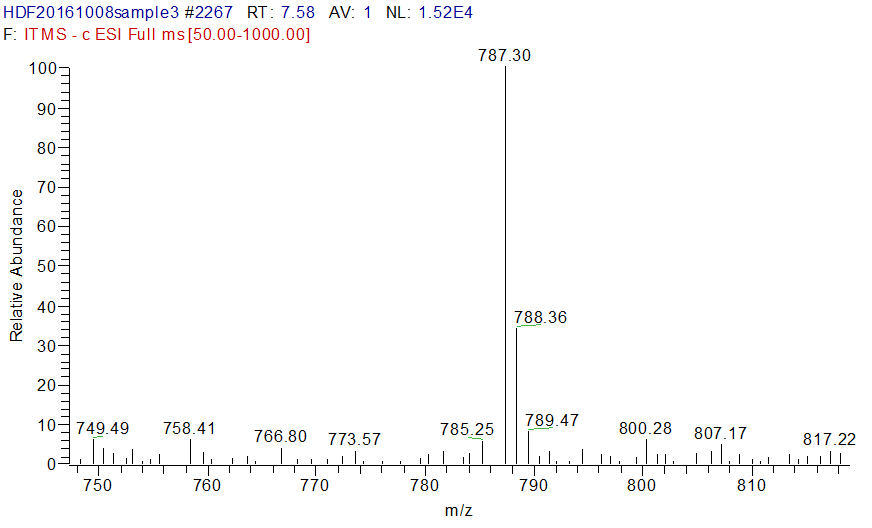


The mass spectrum of quercetin-3-gentiobioside-7-glucoside


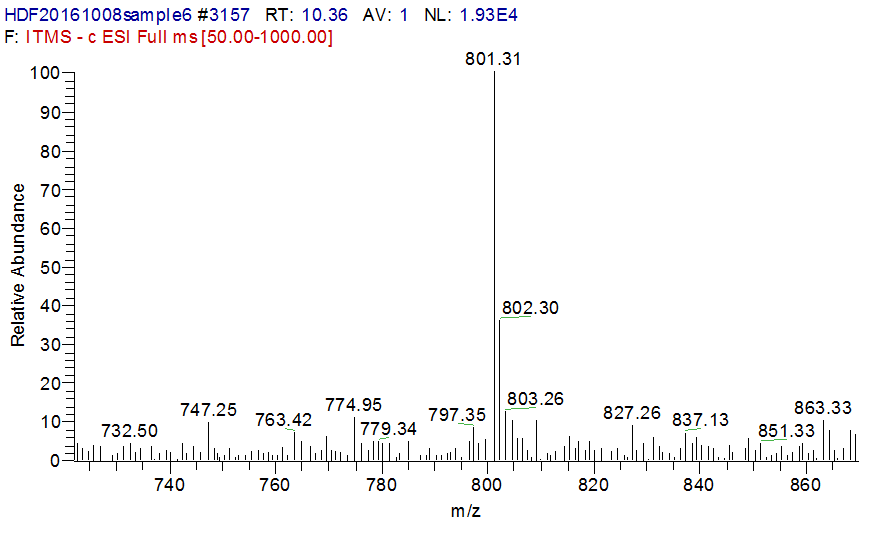


The mass spectrum of isorhamnetin-3-gentiobioside-7-glucoside


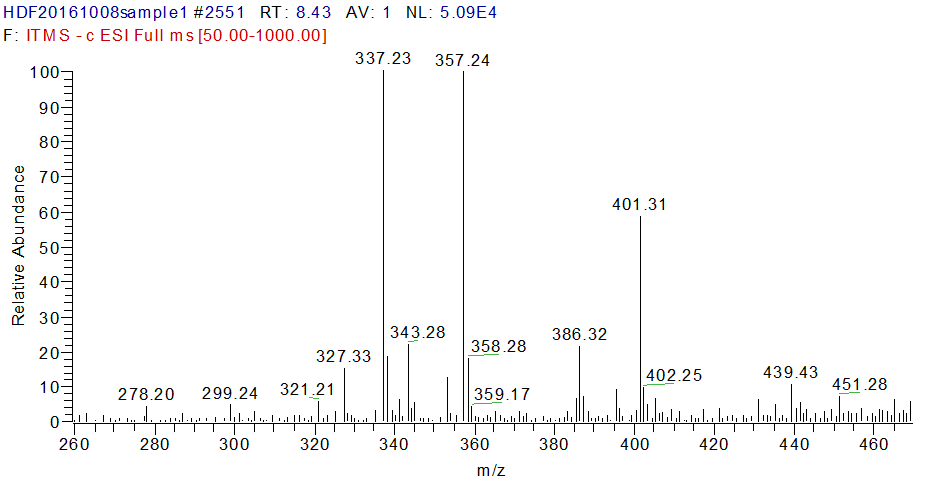


The mass spectrum of 5-p-coumaroylquinic acid


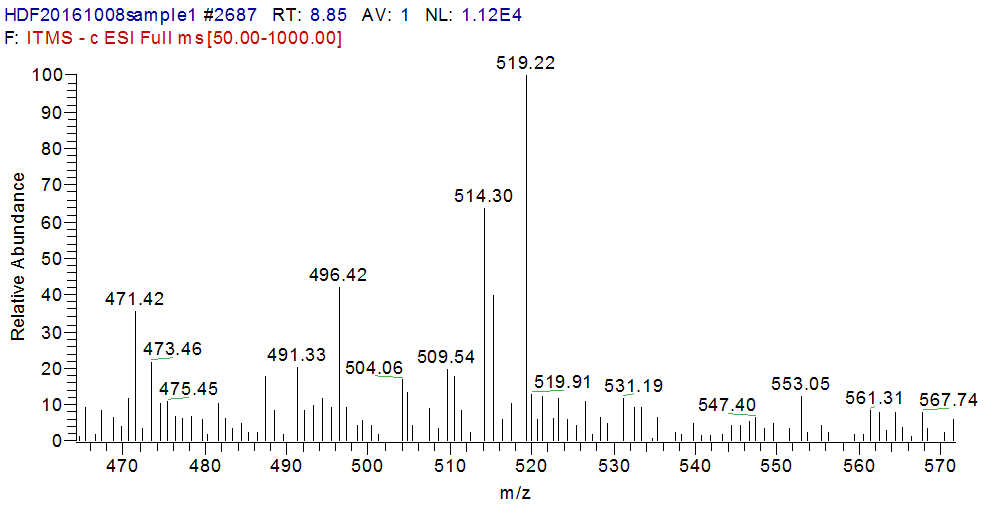
 The mass spectrum of schisantherin D


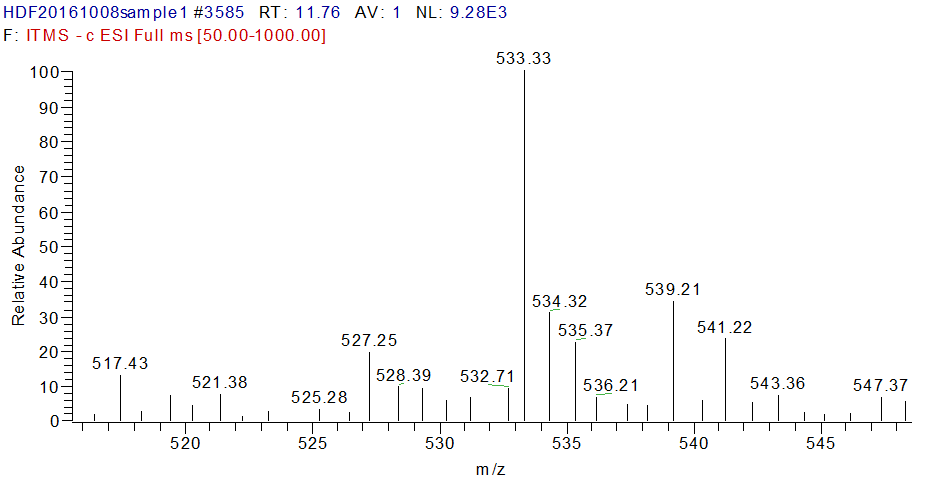


The mass spectrum of phillyrin

S3 Fig: Mass spectra of identified compounds.
